# Mapping the Behavioral Signatures of *Shank3b* Mice in Both Sexes

**DOI:** 10.1007/s12264-024-01237-8

**Published:** 2024-06-20

**Authors:** Jingjing Liu, Jialin Ye, Chunyuan Ji, Wenting Ren, Youwei He, Fuqiang Xu, Feng Wang

**Affiliations:** 1 NMPA Key Laboratory for Research and Evaluation of Viral Vector Technology in Cell and Gene Therapy Medicinal Products, Shenzhen-Hong Kong Institute of Brain Science, Shenzhen Institute of Advanced Technology, Chinese Academy of Sciences, Shenzhen, 518055 China; 2 Shenzhen Key Laboratory of Viral Vectors for Biomedicine, Translational Research Center for the Nervous System, Shenzhen Institute of Advanced Technology, Chinese Academy of Sciences, Shenzhen, 518055 China; 3 Key Laboratory of Quality Control Technology for Virus-Based Therapeutics, Guangdong Provincial Medical Products Administration, Shenzhen Institute of Advanced Technology, Chinese Academy of Sciences, Shenzhen, 518055 China; 4 Shenzhen Key Lab of Translational Research for Brain Diseases, Translational Research Center for the Nervous System, Shenzhen Institute of Advanced Technology, Chinese Academy of Sciences, Shenzhen, 518055 China; 5 Guangdong Provincial Key Laboratory of Brain Connectome and Behavior, the Brain Cognition and Brain Disease Institute, Shenzhen Institute of Advanced Technology, Chinese Academy of Sciences, Shenzhen, 518055 China; 6 CAS Key Laboratory of Brain Connectome and Manipulation, the Brain Cognition and Brain Disease Institute, Shenzhen Institute of Advanced Technology, Chinese Academy of Sciences, Shenzhen, 518055 China; 7 University of Chinese Academy of Sciences, Beijing, 100049 China

**Keywords:** Autism, *Shank3b*, Spontaneous behavior, 3D animal motion-capture system, Computational ethology, Sex differences

## Abstract

Autism spectrum disorders (ASD) are characterized by social and repetitive abnormalities. Although the ASD mouse model with *Shank3b* mutations is widely used in ASD research, the behavioral phenotype of this model has not been fully elucidated. Here, a 3D-motion capture system and linear discriminant analysis were used to comprehensively record and analyze the behavioral patterns of male and female *Shank3b* mutant mice. It was found that both sexes replicated the core and accompanied symptoms of ASD, with significant sex differences. Further, *Shank3b* heterozygous knockout mice exhibited distinct autistic behaviors, that were significantly different from those those observed in the wild type and homozygous knockout groups. Our findings provide evidence for the inclusion of both sexes and experimental approaches to efficiently characterize heterozygous transgenic models, which are more clinically relevant in autistic studies.

**Supplementary Information:**

The online version contains supplementary material available at 10.1007/s12264-024-01237-8.

## Introduction

Autism spectrum disorders (ASD) are a diverse group of conditions characterized by atypical patterns of activities, such as repetitive behaviors, motor dysfunction, anxiety, and some degree of difficulty in social interaction and communication [1, 2]. Recent studies have shown that the prevalence of ASD is increasing, estimated 1 in 44 individuals in the United States. ASD affects males more frequently than females, with a male-to-female ratio of 4:1 [3–6]. Clinically, heterozygous mutations account for most cases underlying ASD patients [7]. The etiology of ASD has been reported to be associated with various genes [8–12], including *Shank3* [13–15]. Plays an important role in the excitatory synapse, whose disruption or deletion leads to autistic-like behaviors [16–18]. *Shank3b* homozygous knockout (KO) mouse is often used as an ASD model due to its self-injurious grooming, abnormal social interactions, and anxiety-like behaviors [16, 19–23]. Additionally, *Shank3* mutations account for approximately 1% of human ASD cases [7]. Therefore, the study of *Shank3* mutant mice can provide valuable insights into various aspects of ASD in humans, including behaviors, neural mechanisms, clinical diagnosis, and treatments.

Spontaneous behavior is an essential indicator for evaluating the basic state of animals. 3D capture systems, which combine video tracking systems and machine learning methods, are more efficiently and comprehensively assessing animals’ behavioral phenotypes [24–29] and have been used to analyze the spontaneous behavior of animal models with ASD [30, 31]. For example, Huang et al. observed that just a small proportion of female *Shank3b* KO mice exhibited a weaker tendency for autistic-like behavior than males. Moreover, their study found no significant difference between male and female KO mice at the group level in terms of spontaneous behavior [30]. Another study revealed that male KO mice display more severe impairments than females in motor coordination [32]. However, there is still a lack of adequate evaluation of spontaneous behavior, especially those involving heterozygous and female mice, which could potentially affect our understanding of animals’ natural state due to insufficient observation in new environments.

In this study, by using a 3D-motion capture system, we analyzed the spontaneous behaviors of both male and female *Shank3b* wild-type littermates (WT), heterozygous (HE), and KO mice, and observed a broader range of behavioral characteristics, including 13 movements and 5 clusters, of *Shank3b* mutant mice. More detailly, *Shank3b* KO and HE mice for both sexes exhibited significantly different spatiotemporal behavioral dynamics compared to WT littermates, with the more prominent of the KO groups. For example, both the HE and KO mice spent significantly less time on walking, stepping, and locomotion, but more time on pausing. As to the movements closely related to ASD, such as grooming and hunching, the WT, HE, and KO groups of both sexes exhibited a gradually increased trend. Based on these characteristics, we can easily discriminate the three groups from each other with line discriminant analysis (LDA). Additionally, significant sex differences existed between the male and female mice within the same genotype. Our studies, including both sexes and three genotypes, not only provide comprehensive behavioral data and empirical support for both sexes in experiments utilizing *Shank3b* mice but also potentially lead to more accurate diagnosis and specific development of effective therapeutic strategies for ASD.

## Material and Methods

### Animals

Adult *Shank3b* heterozygous knockout mice (B6.129-*Shank3*tm2Gfng/J) were purchased from the Jackson Laboratory (Stock No: 017688). To generate wild-type littermates (WT; *Shank3b*^*+/+*^), heterozygotes (HE; *Shank3b*^*+/-*^), and homozygous knockout (KO; *Shank3b*^*-/-*^) mice, 8 to 12-week-old heterozygous males were crossed with age-matched heterozygous females. All mice were housed in cages by genotype, with 3–5 mice per cage, at 20–25°C in a relative humidity of 45%–75%. They were maintained on a 12 h light/dark cycle (lights off at 20:00) and had *ad libitum* access to standard food and water. Behavioral experiments were performed on mice aged between 10–12 weeks. All husbandry and experimental procedures were approved by Animal Care and Use Committees at the Shenzhen Institute of Advanced Technology (SIAT), Chinese Academy of Sciences (CAS) (IACUC number: SIAT-IACUC-20210226-ZKYSZXJJSYJY-RZC-WF-A0923-03). To ensure unbiased analysis, all behavioral experiments were performed and analyzed with experimenters blinded to genotypes. The genotypes of transgenic mice were validated by PCR using Primer mix #1 (to distinguish Mut and WT) and Primer mix #2 (to distinguish KO and HE): The Primer mix #1 forward primer was GAGACTGATCAGCGCAGTTG, and the reverse primer was GCTATACGAAGTTATGTCGAC TAGG. The Primer mix #2 forward primer was GAGACTGATCAGCGCAGTTG, and the reverse primer was TGACATAATCGCTGGCAAAG.

### Spontaneous Behavioral Test

A spontaneous behavioral test was conducted on heterozygous, homozygous *Shank3b* mutant mice, and wild-type littermates of both sexes. Mice were gently introduced into a fixed corner in an open field arena and allowed to explore freely in the box for 60 min. The behavior of each mouse was recorded and analyzed. After each test, the chamber was cleaned with 25% ethanol.

### The 3D-motion Capture System

The 3D-motion capture system was used to capture the movements of *Shank3b* mutant mice during the spontaneous behavioral test. The open-field box is made up of a transparent acrylic wall that stands 30 cm tall and a white plastic square floor with sides measuring 40 cm in length. Although a small cuboid (15 cm in length, 18 cm in width, and 15 cm in height) was present in one corner of the open field box, an acrylic transparent partition was placed at the junction of the two boxes to prevent the mouse from accessing the small cuboid free. The open field arena was positioned at the center of a movable stainless-steel support framework measuring 130 × 130 × 90 cm^3^. The framework had four Intel RealSense D435 cameras mounted orthogonally on its four supporting pillars, and a 56-inch screen was placed horizontally but face down on the top of the shelf to provide uniform and stable white background light. Animals’ behavioral data were extracted from 16 key body parts, including the nose, left ear, right ear, neck, left front limb, right front limb, left hind limb, right hind limb, left front claw, right front claw, left hind claw, right hind claw, back, root tail, middle tail, and tip tail. These key points were used to reconstruct 3D skeletons. The detailed methods, including camera calibration, and animals’ behavioral image acquisition, of this 3D multi-view motion-capture system setup (BA-DC01, Shenzhen Bayone BioTech Co., LTD, Shenzhen), were described in our previous work [30, 33].

### Behavior Atlas Analysis

**Fig. 1:**
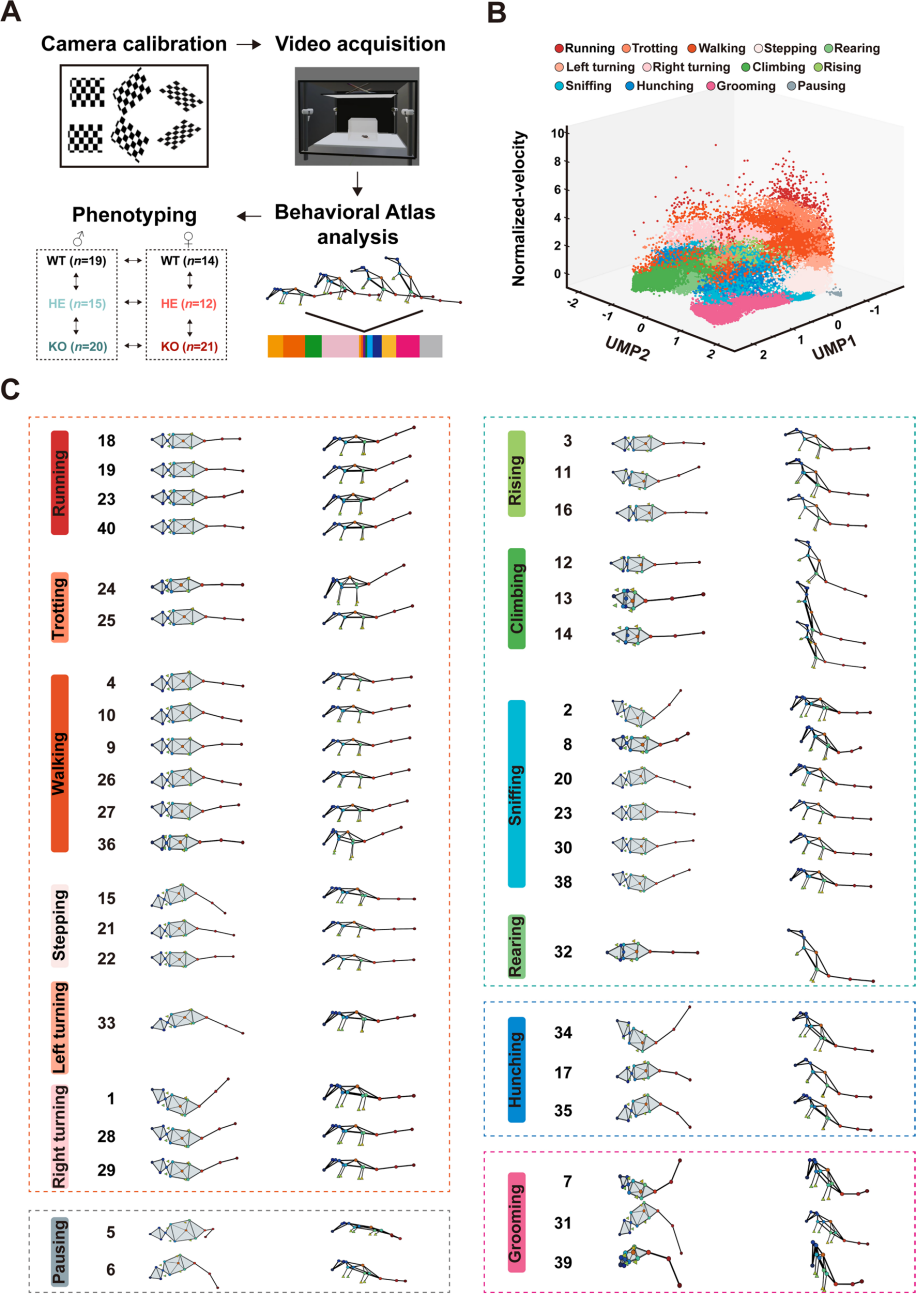
Movement identification of *Shank3b* mutant mice in both sexes. **A** Experimental paradigm for the multi-view video capture system-guided spontaneous behavioral phenotyping in *Shank3b* mutant mice of both sexes. **B** Spatiotemporal feature space of behavioral components. Each dot on the 3D scatter plot represents a movement bout (*n =* 650 bouts), with the thirteen different colors indicating the corresponding thirteen movements. **C** Landscape of thirteen movements, with the average skeleton of all frames within each movement phenotype shown from both the top and the side view.

Complex mammalian behavior typically involves both locomotion and non-locomotion movements. Locomotion can be easily identified using velocity-based parameters, whereas non-locomotory movement, which is controlled by many degrees of freedom, is associated with the movement of limbs or organs rather than the torso. In this study, Behavior Atlas was used to analyze the behavioral data acquired by the 3D-motion capture system. The dynamic time alignment kernel was then used to measure similarity among all non-locomotory movement segments and separate them by different features. Uniform manifold approximation and projection (UMAP) was used to reduce the high-dimensional non-locomotory movement features to 2D ones, and 40 behavioral phenotypes were classified by unsupervised clustering. To identify 40 behavior phenotypes more precisely, we manually checked 50 video segments from each cluster center in the feature space. Behavioral movement was identified when the consistency rate of that phenotype cluster was over 80%. The feature space in Fig. 1B demonstrated good-quality clustering of 13 behavioral movements.

### Supervised Movement Annotation

To identify different types of movement phenotypes in *Shank3b* mutant mice, 100 video clips of each type of movement were randomly selected and manually examined. Similar movements were grouped based on their biological relevance, resulting in the identification of 13 basic types of spontaneous movements. For instance, grooming on different parts of the body (such as the left side, right side, and face) was categorized under a single label grooming. These movements were then further classified into 5 clusters based on their similar behavioral characteristics or specific movements associated with ASD, as detailed in Table 1.

**Table 1:** Definitions of all the movements and clusters

Clusters	Movements	Definition
Locomotion	Running	The mouse locomotes with relatively high-speed
	Trotting	The mouse locomotes at a slow and intermittent pace
	Walking	The mouse locomotes with relatively low speed
	Stepping	The mouse takes a step forward with a short-distance locomotion
	Right turning	The mouse bends its body to the right or turns the body to the right while locomotion
	Left turning	The mouse bends its body to the left or turns the body to the left while locomotion
Exploration	Rearing	The mouse stands on its hind legs with the back straight
	Sniffing	The mouse investigates the environment with the nose held in the air or contacts the environment with the nose closely
	Rising	The mouse rises from four legs on the ground to steadily stand on its hind legs
	Climbing	The mouse rises from four legs on the ground to steadily stand on the wall with its hind legs
Forced posture	Hunching	The mouse stands on its hind legs with the back bent
Maintenance	Grooming	The mouse licks its fur, grooms with the forepaws, or scratches with any limb
Nap	Pausing	The mouse keeps still somewhere without any other locomotion

### Movements Label Correction

To improve the accuracy of movement recognition, some movement labels were validated against specific rules. For example, the movement sniffing with a horizontal velocity of fewer than 15 mm/s and lasting more than 3 seconds was revised to ‘pausing’.

### Discrimination Analysis Across *Shank3b* WT, HE, and KO Mice

LDA is a classical statistical machine-learning method for supervised classification tasks. To discriminate *Shank3b* WT littermates, HE, and KO for both sexes, we used time fractions and frequencies of 13 movements, as well as grooming, total distance traveled in the open field arena, time spent in the traditional center and perimeter area, and characteristics of clusters, respectively by LDA.

### Temporal Variation of 13 Movements and/or Clusters

To investigate the temporal variation of each movement, we identified every movement and calculated the time fractions and frequencies of each specific movement and/or clusters in every minute with the formula $T_{i} = \frac{{M_{i} }}{{M_{Total} }}, i \in \left[ {1,60} \right]$. The comprehensive percentage of each minute of movement and/or cluster was calculated by averaging the values of all mice in the same group.

### Cluster Transition Probability

For cluster transition probability analysis, each cluster was considered as a state in the probabilistic graphical model [29]. Each cluster fragment, regardless of its interval, was treated as an event and the probabilities of states were calculated based on the number of cluster events. State transition probabilities were calculated based on the frequency of the previous cluster event transformed to the next cluster event. To evaluate the differences in transition probabilities between different groups, we calculated the average differences in transition probability from cluster one to another. Those with a statistically significant *P <* 0.05 transition probability were excluded (Fig.6E, F, I, J).

### Area Division

To accurately determine the boundary between the center and perimeter area in the open field arena, we used the residence time points of the mouse’s back based on the animal’s actual trajectory [29]. We established a consistent coordinate system with the central point of the box as the origin (0,0), and the four corner points calibrated to (−200, 200), (−200, 200), (200, 200), and (−200, −200). Then, we defined a square frame with a side length labeled as ‘a’ that expanded incrementally from the center to the perimeter, to count the residence time points of the mouse’s back within the arena. The growth curve corresponded with the varied residence time points of the mouse's back in the enlarging square frame (Fig. 4A, bottom). The boundary lines were defined by the square frame that lay within 10% of the growth curve's peak prominence. This data-driven method provided enhanced precision in dividing the center and peripheral area based on the animal’s actual trajectory.

### Statistical Analysis

All comparisons between groups were made using littermate animals with experiments performed at the same time. The behavioral experiments and data analyses were conducted double-blind. The data were analyzed using Prism 9 (GraphPad 9.0) and presented as means ± SEM, as noted in the figure legends. For the comparison of 13 different movement types and 5 different clusters among WT, HE, and KO mice for both sexes in the spontaneous behavioral test, two-way analysis of variance (ANOVA) was used, Turkey multiple comparisons test was applied as a *post hoc* analysis. To analyze the time percentage in the different areas among WT, HE, and KO mice of both sexes, we used two-way ANOVA followed by the Dunnett post hoc multiple comparisons test for the analysis of area preference. Statistical analyses of the comparison of different movements and clusters between male and female mice were determined by two-way ANOVA followed by the Turkey *post hoc* multiple comparisons test. One-way ANOVA was used in the statistical analyses of the total distance traveled in the open field box of both sexes. Statistical significance was denoted by ***P < 0.001, **P < 0.01, *P < 0.05.

## Results

### Movements Identification by a 3D-Motion Learning Framework

To assess the spontaneous behavior patterns and characteristics of *Shank3b* mutant mice, we employed a 3D-motion capture system and Behavioral Atlas software [7] (Fig. 1A) to collect and analyze behavioral data from 114 *Shank3b* mutant mice aged between 12–16 weeks (male: KO = 20, HE = 15, and WT = 19; female: KO = 21, HE = 12, and WT = 17) from both sexes. To accurately capture the motion of different parts of the body, we tracked 16 key body parts to reconstruct the skeleton of a mouse. Using unsupervised clustering, 40 types of behavioral movements were identified. Then, we manually designated 13 types of major behavioral movements based on highly similar postures, including running, trotting, walking, right turning, left turning, stepping, rearing, climbing, hunching, rising, sniffing, grooming, and pausing. Behavioral atlas analysis effectively revealed the structure of the spontaneous behavior in *Shank3b* mutant mice (Fig. 1B, C) (Table 1).

### Different Genotypes Exhibit Different Spontaneous Behavioral Characteristics in Both Sexes

**Fig. 2:**
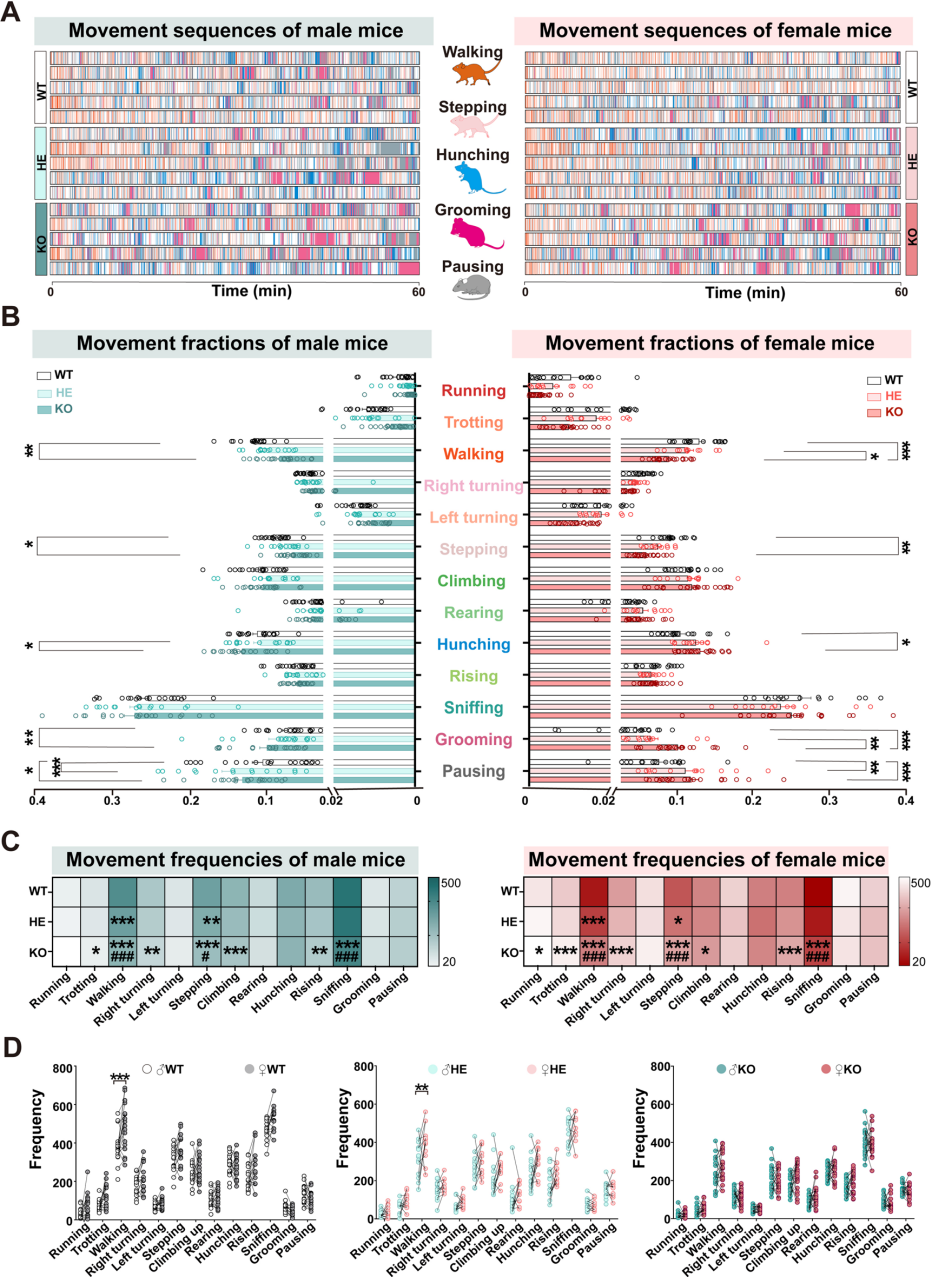
Distinct spontaneous behavioral characteristics of *Shank3b* mutant mice in both sexes over 60 min. **A** Representative ethogram of the five significant different movements among *Shank3b* WT, HE, and KO mice of both male (left) and female (right) mice (Up, WT; Middle, HE; Down, KO). Five animals were randomly selected in each group. Middle color-coded images correspond to the five different movements, top to bottom: walking (orange), stepping (light pink), hunching (blue), grooming (rose red), and pausing (gray). **B** Comparison of the fraction of thirteen movements among *Shank3b* WT, HE, and KO mice (Left: male; KO, deep green; HE, pale green; WT, blank; Right: female; KO, dark red; HE, pink; WT, blank). Middle color-coded labels indicate the movements. Statistics: two-way ANOVA followed by Turkey post hoc multiple comparisons test. **C** Comparison of the frequency of thirteen movements among *Shank3b* WT, HE, and KO mice (Left: male; Right: female). Statistics: two-way ANOVA followed by Turkey post hoc multiple comparisons test. **D** Comparison of the frequency of thirteen movements between male and female *Shank3b* mutant mice (left: WT; Middle: HE; Right: KO). Statistics: two-way ANOVA followed by Turkey post hoc multiple comparisons test. ***P < 0.001, **P < 0.01, *P < 0.05.

To analyze the spontaneous behavioral repertoires of *Shank3b* WT, HE, and KO mice, we examined the behavioral sequences (Fig. 2A, Fig. S1A, B) and analyzed time fractions (Fig. 2B) and frequencies (Fig. 2C) of all the movements in the three groups for both sexes over 60 min. Our findings revealed that walking and stepping, which may represent motor deficit function in patients, showed significant differences across the three groups for both sexes (Fig. 2C), and KO mice of both sexes exhibited a significant reduction in other movements such as trotting, right turning, climbing, rising, and sniffing, which may represent the exploratory function in patients, but an increase in grooming, which may represent the stereotyped movements in patients, compared to their WT counterparts. Female KO mice also showed significantly reduced running and increased hunching, which may represent forced posture in patients, compared to the WT group. Interestingly, HE mice from both sexes showed a significant decrease in walking, stepping, and sniffing compared to the KO mice, coupled with a pronounced increase in pausing compared to WT mice and in grooming compared to female KO mice. Furthermore, we evaluated sex differences in all movements across the three groups and found that the frequency of the walking exhibited significant differences between sexes within each genotype (Fig. 2D, Fig. S1C). Specifically, the WT groups displayed the most pronounced differences, followed by the HE group, while the KO group revealed no noticeable differences.

Besides, the total travel distance in the open field arena showed that HE and KO mice of both sexes exhibited a significant decrease in walking compared to the WT group (Fig. S2), with the KO group decreasing most prominent. No significant differences were observed between male and female mice of the same genotype group (Fig. S2E–G). Overall, our findings indicated that different genotypes of *Shank3b* mutant mice exhibited diverse movement characteristics across both sexes.

### Genotypes Show Distinct Temporal Patterns of Spontaneous Behaviors

**Fig. 3:**
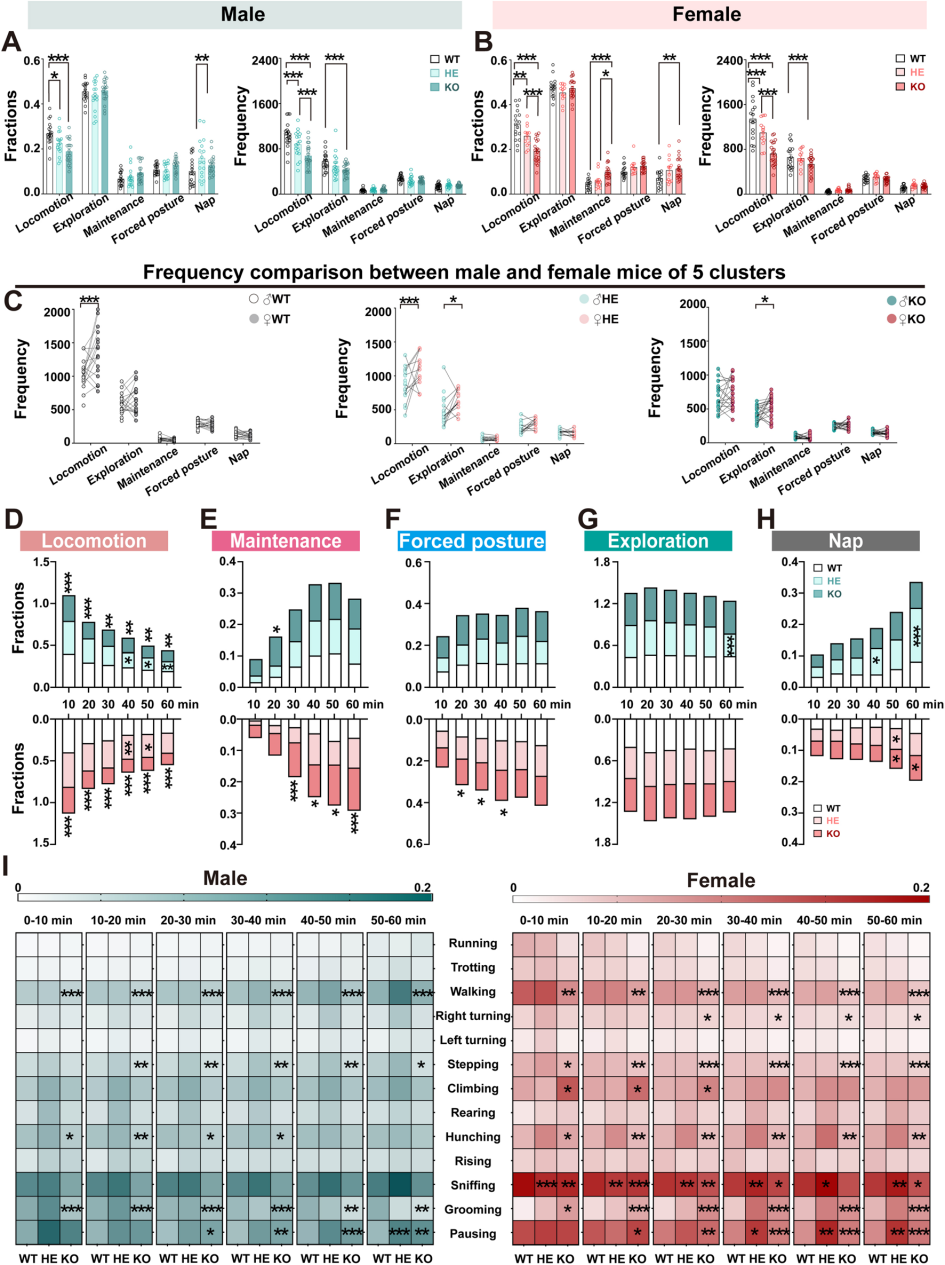
Temporal dynamics of clusters exhibited by both male and female *Shank3b* mutant mice. **A–B** Comparison of the time fractions and frequency of five clusters among male (**A** left: fractions; Right: frequency) and female (**B** left: fractions; Right: frequency) *Shank3b* WT, HE, and KO mice over 60 min. Statistics: two-way ANOVA followed by Turkey post hoc multiple comparisons test. All data are presented as means ± SEM. **C** Comparison of the frequency of thirteen movements between male and female *Shank3b* mutant mice (left: WT; Middle: HE; Right: KO). Statistics: two-way ANOVA followed by Turkey post hoc multiple comparisons test. ***P < 0.001, **P < 0.01, *P < 0.05. **D–H** Temporal dynamics of the five clusters in male and female *Shank3b* mutant mice over 60 min, with the sequence from D to H representing locomotion, maintenance, forced posture, exploration, and nap. The upper part of the panels shows the results for male mice (KO, deep green; HE, pale green; WT, blank), while the lower part shows the results for female mice (KO, dark red; HE, pink; WT, blank). Statistics: two-way ANOVA followed by Dunnett post hoc multiple comparisons test. All data are presented as means ± SEM. **I** Temporal dynamics of the 13 movements in every 10-min interval of male (left) and female (right) *Shank3b* mutant mice. Statistics: two-way ANOVA followed by Dunnett post hoc multiple comparisons test. ***P < 0.001, **P < 0.01, *P < 0.05.

To better understand the functional behavior characteristics of *Shank3b* WT, HE, and KO mice, we classified 13 movements into 5 clusters based on their similarities or specific links to ASD, including locomotion, exploration, maintenance, forced posture, and nap. Similarly, we analyzed the time fractions and frequencies of each cluster across both sexes over 60 min. Our results showed that time-frequency among all three groups for both sexes showed significant differences in locomotion, with the KO group of the most prominent, mainly attributed to walking and stepping (Fig. 3A, B, I). Further, KO mice exhibited a significant decrease in exploration of both sexes and prominent increases in maintenance and nap for the female group, probably resulting from the increased climbing, grooming, pausing, and decreased sniffing (Fig. 3A, B, I). Additionally, sex differences in the movement fractions (Fig. S3) and frequencies across time (Fig. 3C) of each cluster were examined across the three genotypes. We found that locomotion and exploration of both sexes exhibited significant differences among the three groups. Subsequently, we divided the observational 60-min period into six 10-min segments and conducted an in-depth analysis of the temporal patterns of each cluster (Fig. 3D-H) and movement (Fig. 3I) for both sexes (Fig. S3, S4). Our results showed that both male and female KO mice allocated less time for locomotion cluster across all 10-minute segments, particularly during the last three segments for male HE mice compared with the WT (Fig. 3D). Female KO mice spent significantly more time in the maintenance cluster from the third segment onwards but were only limited to the second segment for the male KO group (Fig. 3E). The notable increase in time spent on the nap cluster of female KO and male HE groups in the whole 60-min period was largely attributed to the significant increase during the last two segments for females and the fourth and final segments for males, respectively (Fig. 3H). No noticeable differences in exploration and forced posture were found in each segment except between the second to fourth segments for the female KO group (Fig. 3F, G). Besides, we conducted a time-based comparison of sex differences in the time spent on the 5 clusters (Fig. S5) and 13 movements between male and female *Shank3b* WT (Fig. S6), HE (Fig. S7), and KO (Fig. S8) mice. We found that throughout the entire duration, as well as across all six 10-minute segments, there were virtually no significant sex differences observed, indicating a mild sex-dependent variation in the temporal behavioral characteristics among the three groups. These findings provide further insights into different temporal behavioral characteristics among the three groups of *Shank3b* mutant mice of both sexes.

Additionally, we explored the temporal characteristics of the distance traveled by the six groups. Although there was a significant increase in the total distance traveled across all groups and both sexes across all segments, we found a significant decline at each segment for the KO groups and during the last two segments for the HE groups compared to the WT groups (Fig. S9A–D). Notably, the distance traveled in each segment was significantly decreased over time among the three groups, with the most drastic decline being in the KO groups across all segments, while only the fourth and fifth for the male HE groups and the third to fifth segments for female HE groups compared with the WT group, respectively (Fig. S9E–H).

### Spatial Preferences in the Open Field Vary Among Different Genotypes

**Fig. 4:**
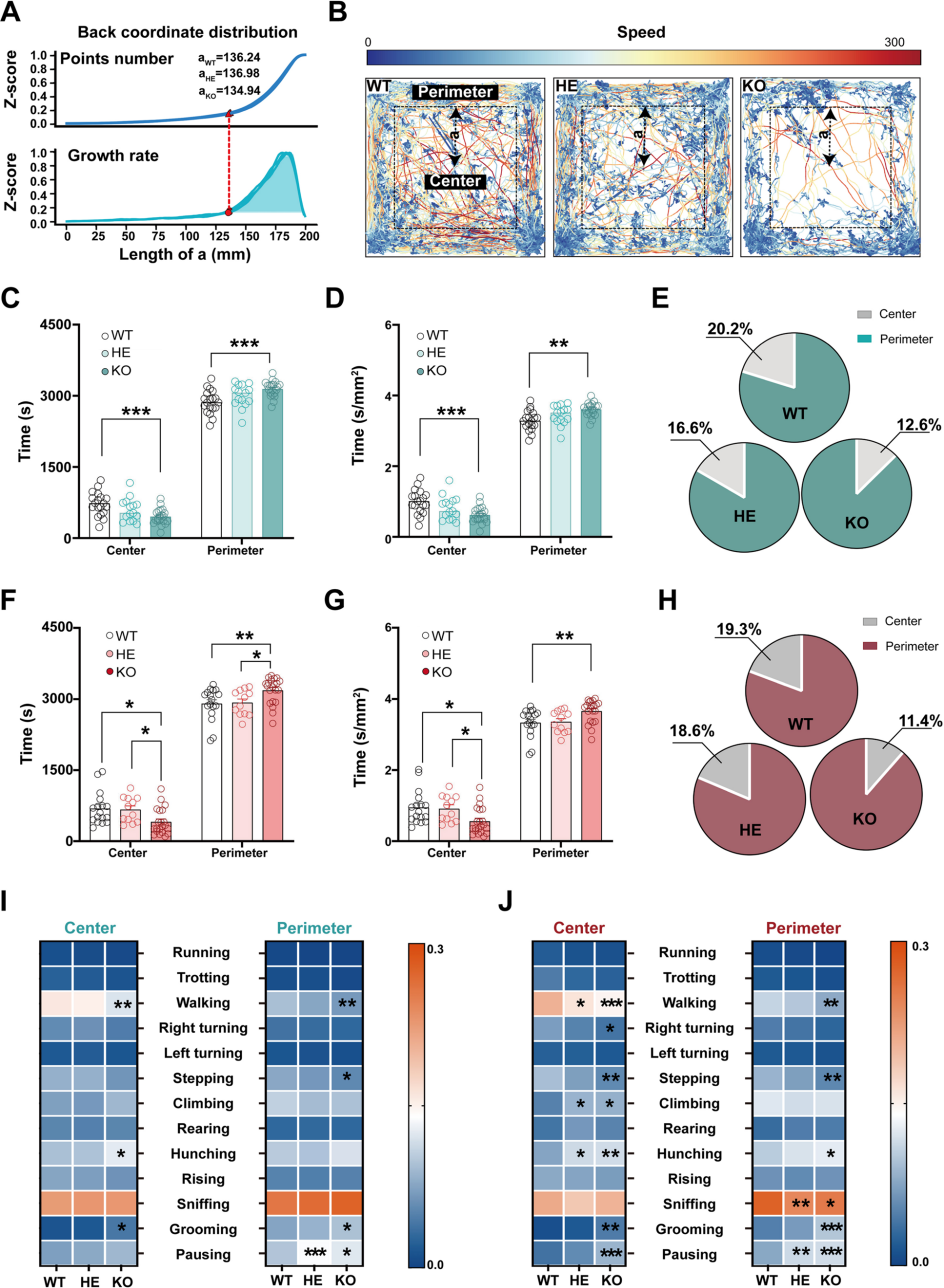
Spatial preference characteristics of movements exhibited by male and female *Shank3b* mutant mice. **A** Density distribution curve with back coordinates of three *Shank3b* mutant mice, the red triangle denoting the starting point of the 90% peak prominence as a division of the heterogeneity occupancy density regions. **B** Representative images of the spatial preference of three *Shank3b* mutant mice, with the dotted lines delineating the boundary between the center and the perimeter. **C, F** Comparison of the time fractions in the center and the perimeter among male (**C**) and female (**F**) *Shank3b* WT, HE, and KO mice in 60 min. **D**, **G** Comparison of the time fractions per unit area in the center and the perimeter among male (**D**) and female (**G**) *Shank3b* WT, HE, and KO mice in 60 min. **E**, **H** The pie diagrams compare the time percentage of the center and perimeter area for male (**E**) and female (**H**) *Shank3b* WT, HE, and KO mice. **I** Comparison of movement variation in the center (left) and perimeter (right) areas for male *Shank3b* WT, HE, and KO Mice Over a 60-min duration. **J** Comparison of movement variations in the center (left) and perimeter (right) areas for female *Shank3b* WT, HE, and KO mice over a 60-min duration. ***P < 0.001, **P < 0.01, *P < 0.05.

The spatial preferences in the open field test are commonly used to evaluate the anxiety-like behavior in rodents. Typically, the center area is defined as half the length of the open field, and decreased time spent in the center is considered as an increase in anxiety-like level, as shown in *Shank3b* KO male mice [34, 35]. With the new division method, we found that the length of the square boundaries (‘a’, 136.24 mm, 136.98 mm, and 134.94 mm for WT, HE, and KO mice, respectively) did not differ significantly among WT, HE, and KO mice (Fig. 4A, B). Therefore, we standardized the length at 135 mm for subsequent analysis. The time spent in different areas and types of movements therein were evaluated for *Shank3b* mutant mice using both traditional and data-driven region division methods. Comparative analyses of two divisions showed that the KO mice of both sexes spent less time in the central area but more time in the perimeter area compared to the WT group (Fig. 4C–H). The same analysis revealed that male KO mice had decreased walking in the centre and decreased pausing in the perimeter area, while female KO mice had decreased walking, stepping, and right turning in the centre and increased hunching, grooming, and pausing in the perimeter area based on the new division. However, only grooming movement significantly increased in the perimeter area in male KO mice, but no noticeable movement significantly decreased in the center area by the traditional division. These findings revealed that our data-driven method is more compatible with the animal's actual trajectory than the traditional ones, suggesting our region division method provides a more detailed insight into behaviors.

### Different Genotypes Display Distinct Behavioral Transition Patterns

**Fig. 5:**
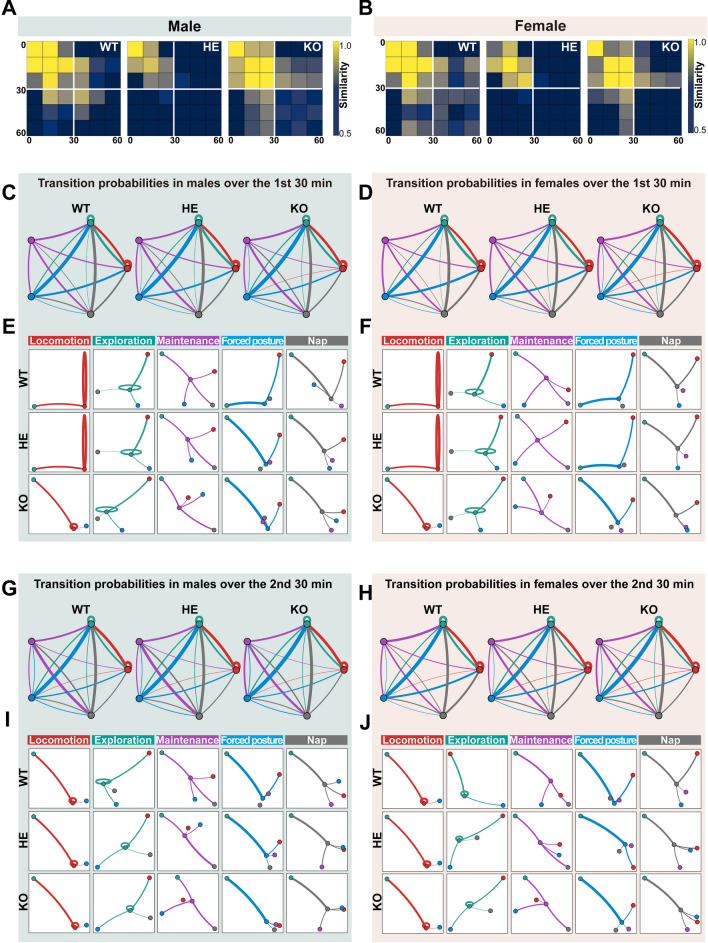
Behavioral transition probabilities of five clusters in male and female *Shank3b* mutant mice. **A, B** Behavioral similarities between the first and the second 30-min duration for male (**A**) and female (**B**) *Shank3b* mutant mice (Left: WT; Middle, HE; Right: KO), with each cell represented the overall similarity of each group within the corresponding timeframes. **C, D, G, H** Behavioral transition probabilities during the first and the second 30 min across the five clusters in male (first: **C;** second: **G**) and female (first: **D;** second: **H**) *Shank3b* WT (left), HE (middle), and KO (right) mice. The five color-coded circles denote the five corresponding clusters. The size of each circle indicates the relative occurrence probability of each cluster, with larger circles denoting higher probabilities. The connecting lines among every two circles represent the transition probabilities of the two clusters, with greater line thickness indicating a higher transition rate. Lines around the circles indicate the self-transition probability based on the movements grouped into the clusters. **E, F, I, J** Connection properties of each cluster in betweenness centrality for both male (first: **E;** second: **I**) and female (first: **F;** second: **J**) *Shank3b* WT, HE, and KO mice during the second 30 min. The magnitude of transition probabilities is symbolized by the length and/or thickness of the lines linking two nodes, with greater length and/or thickness denoting higher probabilities. All lines representing transition probabilities of less than 0.05 are intentionally omitted.

Next, we investigated the influence of the genotypes and sexes on the behavioral temporal sequence of *Shank3b* mice, we analyzed the transition patterns within clusters over an hour. The results showed that the most significant changes and differences among the three groups in both sexes in transition patterns were primarily observed during the first half (Fig. 5A, B). We observed a gradual increase in mutual transition between maintenance and nap across the groups from WT to HE to KO, of both sexes, while the opposite trends were observed between maintenance and locomotion, as well as between forced posture and locomotion (Fig. 5C, D). Moreover, to elucidate the significance of clusters in a behavioral transition network, we computed two-time segments. Our data showed that, during the initial time segment, clusters for both sexes functioned as transitional hubs, manifesting distinct transition patterns when compared to those observed in the WT group. In addition in the HE group, forced posture and nap in males, along with maintenance in females similarly displayed divergent transition patterns relative to WT mice. For example, both the male and female KO mice could transition from locomotion to exploration, as well as to hunching, while locomotion will only transform to exploration in WT mice (Fig. 5E, F). Consistent with the transition patterns of each cluster in the initial time segment, the transition pattern for each cluster as a transitional hub was less pronounced than that in the latter 30-min period (Fig. 5G–J). Overall, mice exhibited relatively stable behavioral connections and transitional patterns, yet transition strategies and behavioral organization of KO and HE groups varied from the WT group.

### Characterization Data Models Distinguish among Different Genotypes

**Fig. 6:**
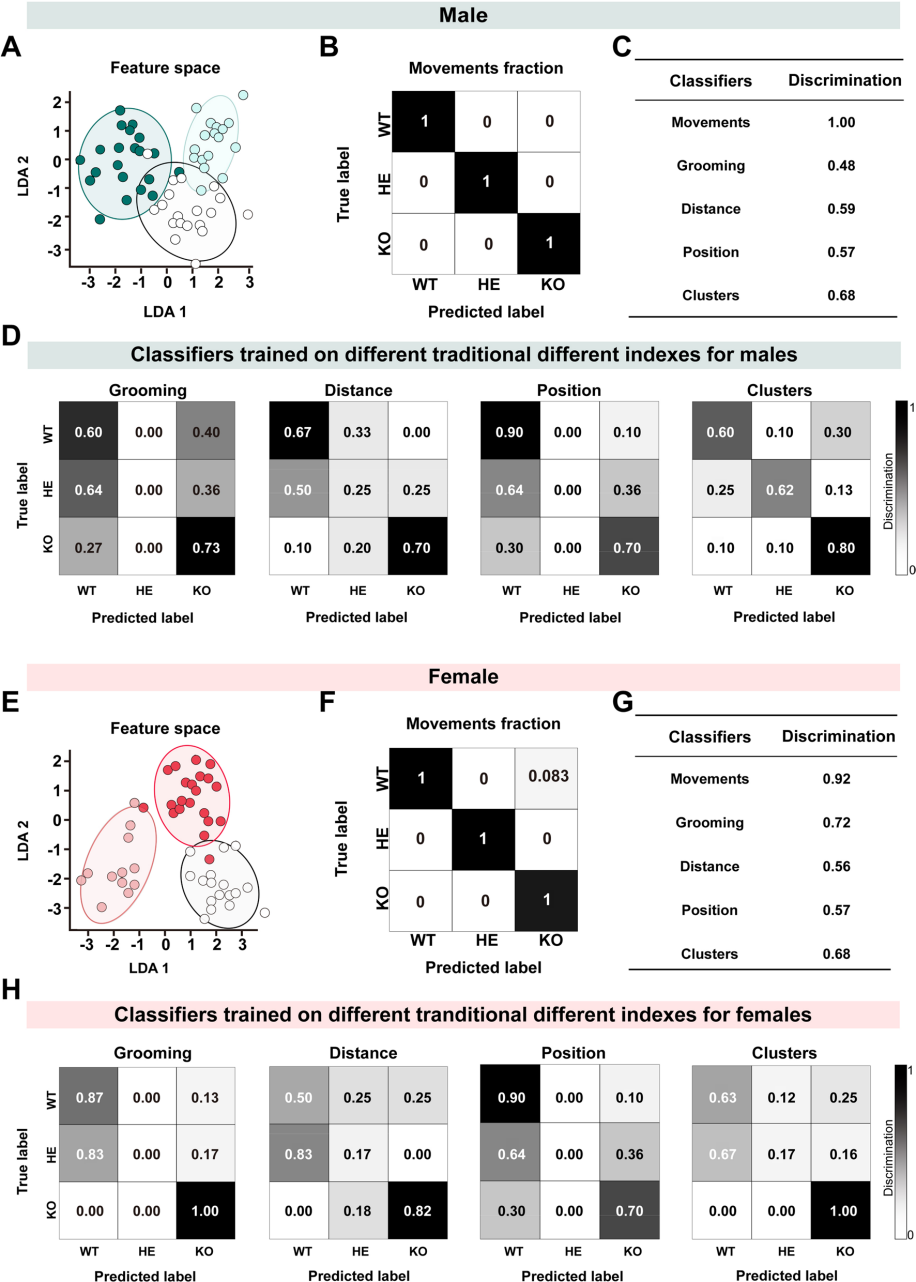
Behavioral dissimilarity among *Shank3b* WT, HE, and WT across both sexes. **A, E** Low-dimensional representation of the thirteen movements in male (**A**) (WT, blank; HE, pale green; KO, deep green) and female (**E**) (WT, blank; HE, pink; KO, dark red) *Shank3b* mutant mice using LDA linear model. **B, F** Normalized classification matrices by all thirteen movements (across rows and columns) for distinguishing male (**B**) and female (**F**) *Shank3b* WT, HE, and KO mice. The numerical values within the matrices represent the count of true and/or predicted instances for each group. The color bar is shared by all matrices. The values, ranging from 0 (depicted in white) to 1 (depicted in black) indicate increasing discrimination. An ideal classifier performance corresponds to a diagonal black with otherwise white fields (Discrimination of 1). **C, G** The discrimination of different classifiers for male (**C**) and female (**G**) *Shank3b* WT, HE, and KO mice. **D, H** Normalized classification matrices by traditional classifiers for distinguishing male (**D**) and female (**H**) *Shank3b* WT, HE, and KO mice.

We explored the feasibility of distinguishing autism-associated mice from WT mice based on the altered spontaneous behavioral patterns exhibited by *Shank3b* WT, HE, and KO mice. LDA was employed to integrate and analyze the behavioral data of all 13 movements. As expected, *Shank3b* mutant mice were largely separated from distinct genotypes for both sexes (Fig. 6A, E). We then trained several other LDA classifiers on different parameters that were traditionally applied to discriminate autism, such as grooming, traveling distance, position in the arena, and clusters, to predict genotypes of different groups (Fig. 6B, D, F, H). Our results revealed that prediction accuracy of the classifier based on movement fractions and frequency (discrimination: male, 1.0; female, 0.92) outperformed the classification based on grooming (discrimination: male, 0.48; female, 0.72), distance (discrimination: male, 0.59; female, 0.56), position in the open field arena (discrimination: male, 0.57; female, 0.57), and clusters (discrimination: male, 0.68; female, 0.68) in identifying autistic from WT animals (Fig. 6C, G). This highlights that the classifier trained on 13 movements exhibited more critical insights compared with other behavioral parameters used in most previous methods.

## Discussion

In this study, we utilized a 3D-motion capture system to investigate the dynamic characteristics of spontaneous behaviors in *Shank3b* mutant mice of both sexes. Our results showed that behavioral signatures were significantly different from each other within the same sex among the three groups but more prominent in KO, and across sexes within the same genotype. Further, these three genotypes can be easily discriminated from each other with the LDA approach for both sexes. By including the female mice and HE mice in both sexes, our study provides more comprehensive behavioral data of *Shank3b* mutant mice, thereby potentially enhancing the efficiency of translational research in ASD.

Although homozygous mouse studies are important for understanding the physiological role of a gene, most human *Shank3b* patients with clinical conditions are heterozygous mutations. This situation present a great challenge historically to separate from the traditionally developed individuals [36–40]. In this study, By integrating the 3D-motion capture system and LDA algorithm, it is feasible to distinguish HE mice from the WT and KO mice for both sexes (Fig. 6). The reasons were probably due to this advanced system being able to capture a broader spectrum of movements [29, 30], as well as the application of LDA to integrate these movement differences among the three groups. More detailly, ASD patients, including rodents with *Shank3* mutations [14, 15, 40], are frequently reported with motor dysfunction [15, 41–43]. In our study, we found that compared with the WT littermates, the HE mice demonstrated a significant increase in pausing but a marked decrease in walking and stepping on movement level, as well as locomotion on cluster level, across both sexes. Given the predominance of mutations in clinical ASD cases, identifying HE mice from WT and KO mice allows a more subtle investigation into the pathogenesis with a animal model more relevant to ASD patients, which enhances the potential for developing more precise and effective therapeutic interventions for ASD.

Most previous studies on patients [44] or mouse models of ASD [45, 46] have usually excluded females, probably due to the sex disparity in prevalence. In our study, we examined equal numbers of both male and female mutations and found significant sex differences across the three groups. Specifically, female HE mice showed a significant increase in walking and locomotion than male HE mice, but the difference was smaller than that between the WT groups, while no significant difference between the KO groups (Fig. 2, 3). Similarly, significant sex differences in exploration between male and female mice were observed in both the HE and KO groups, but not from the WT groups (Fig. 3). Our findings showed that different sexes have different behavioral phonotypes in autistic models, suggesting that females should be included in future mechanistic studies and interventional development.

Given the current clinical reliance on behavioral symptoms for ASD diagnoses, acquiring more comprehensive and accurate behavioral phenotypes is of great importance. For example, repetitive behavior deficits, as one of the core symptoms of autism [14–16, 47–49], are frequently reported in ASD patients, including those with *Shank3* mutations [14, 15, 40]. The self-grooming activity of rodents has been widely employed as an index for repetitive behavior. Our 3D method exhibits significant advantages over traditional 2D approaches to the analysis of grooming behavior. In particular our method not only was able to quantify the fractions and frequencies of grooming, but also provide the spatial information of this movement, highlighting a benefit for accurate evaluation of autism-like behaviors. Moreover, as spectrum disorders, no single model can fully mimic the complexity of the human *Shank3*-related behavioral phenotypes. Hence, incorporating the analysis of social behavior and other different *Shank3*-related mutations with the 3D-motion capture system will help to establish a more robust behavioral dataset. Additionally, further optimizations in algorithms and improvements in spatiotemporal resolution of movements with this system will significantly enhance our insights into *Shank3*-associated ASD animals and other disease models. In conclusion, a more detailed understanding of genotype-phenotype correlation in ASD animal models across both sexes will shed light on the neuropathological mechanism of *Shank3*-associated ASD patients.

### Supplementary Information

Below is the link to the electronic supplementary material.

Supplementary file1 (PDF 4670 kb)
