# Supplementary material for: Mapping the Behavioral Signatures of Shank3b Mice in Both Sexes
Source: Neurosci Bull. 2024 Jun 20;40(9):1299–314. doi: 10.1007/s12264-024-01237-8 (PMC11365888; doi:10.1007/s12264-024-01237-8)
Supplement: Supplementary file 1 — Supplementary file1 (PDF 4670 kb) [file 12264_2024_1237_MOESM1_ESM.pdf]

Supplementary Figures

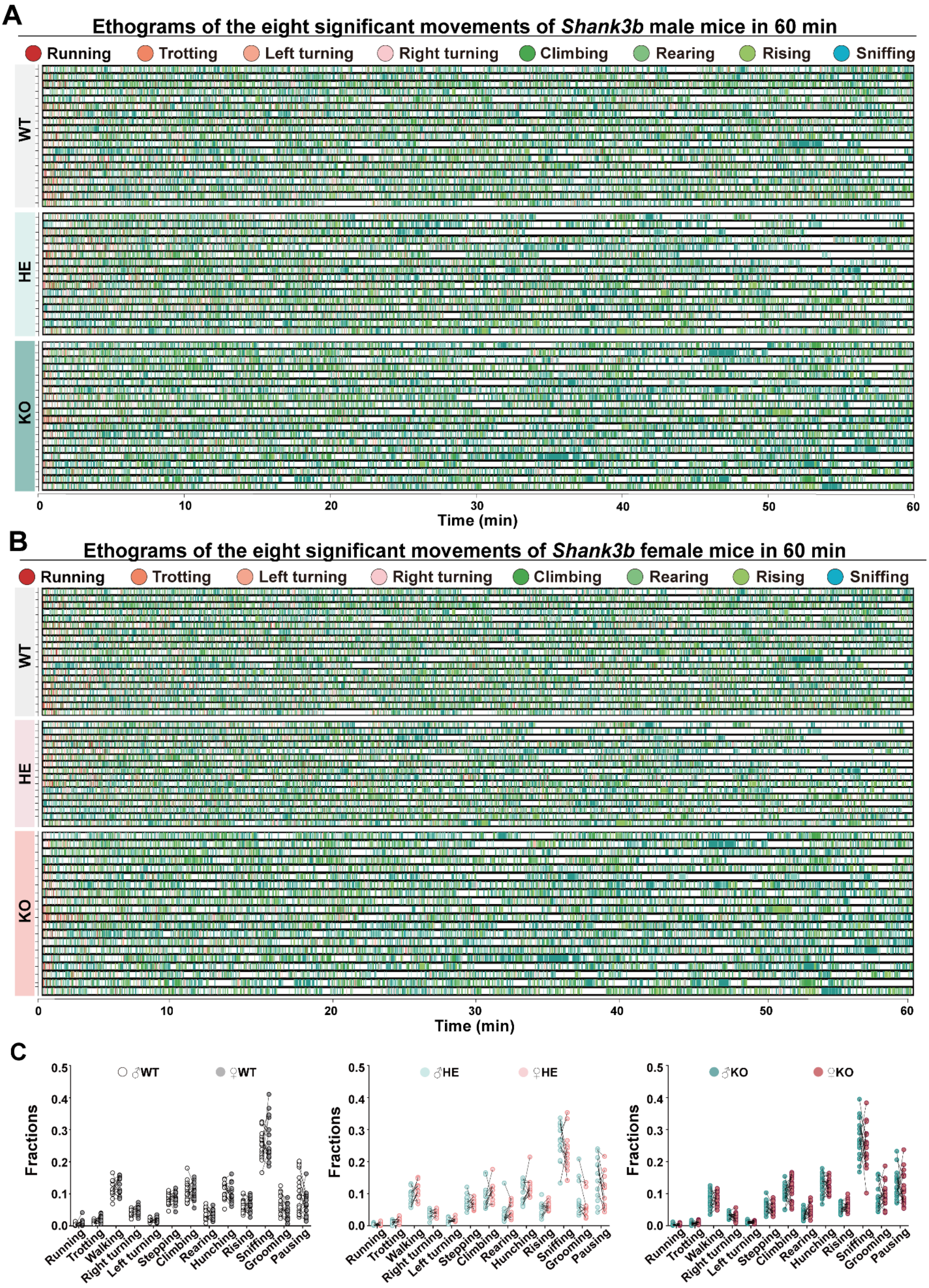

Fig. S1 Spontaneous behavioral characteristics of *Shank3b* mutant mice in 60 min of both sexes. **A, B** Representative ethograms of the eight movements with no difference in male (**A**) and female (**B**) *Shank3b* mutant mice (Up, KO; Middle, HE; Down, WT) in 60 min. **C** Comparison of the fractions of thirteen movements between male and female *Shank3b* mutant mice (left: WT; Middle: HE; Right: KO). Statistics: two-way ANOVA followed by Turkey post hoc multiple comparisons test.

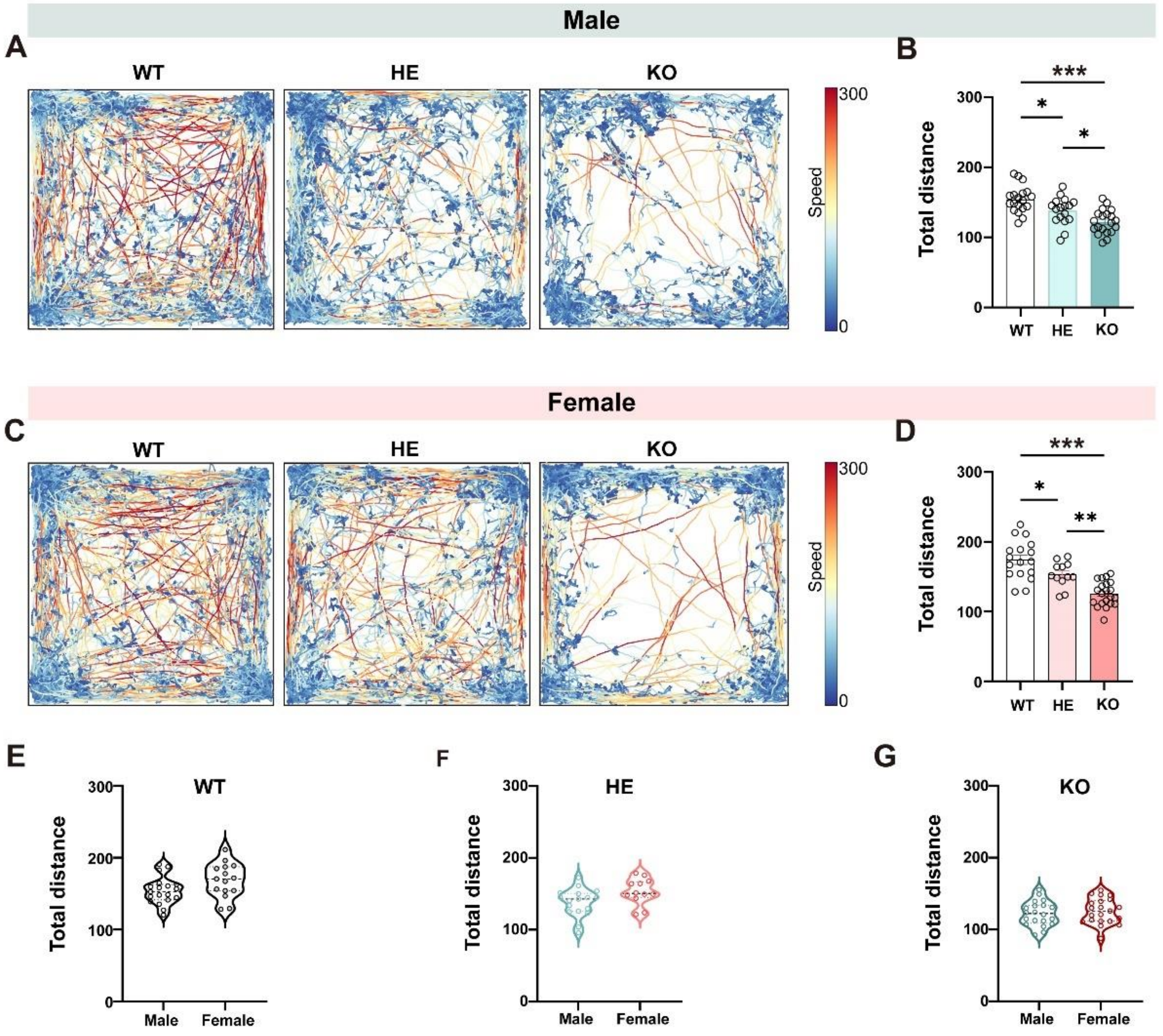

Fig. S2 Different motor characteristics of *Shank3b* WT, HE, and KO mice with both sexes. **A** Representative trajectory images traveled in an open field arena with different speeds of male *Shank3b* WT (left), HE (middle), and KO (right) mice over a 60-min duration. The color gradient, ranging from blue to red represents an increasing speed. **B** Comparison of the total distance traveled in the open field arena of male *Shank3b* WT (left), HE (middle), and KO (right) mice over a 60-min duration. Statistics: ordinary one-way ANOVA followed by Turkey's test. Data are expressed as mean ± SEM. **C** Representative trajectory images traveled in an open field arena with different speeds of female *Shank3b* WT (left), HE (middle), and KO (right) mice over a 60-min duration. The color gradient, ranging from blue to red represents an increasing speed. **D** Comparison of the total distance traveled in the open field arena of female *Shank3b* WT (left), HE (middle), and KO (right) mice over a 60-min duration. Statistics: ordinary one-way ANOVA followed by Turkey's test. Data are expressed as mean ± SEM. **E-G** Comparison of the total distance traveled in the open field arena between male and female mice for *Shank3b* WT (**E**), HE (**F**), and KO (**G**) mice over a 60-min duration. Statistics: two-sided unpaired T-test. Data are expressed as mean ± SEM. ***$P < 0.0001$, ** $P < 0.01$, * $P < 0.05$.

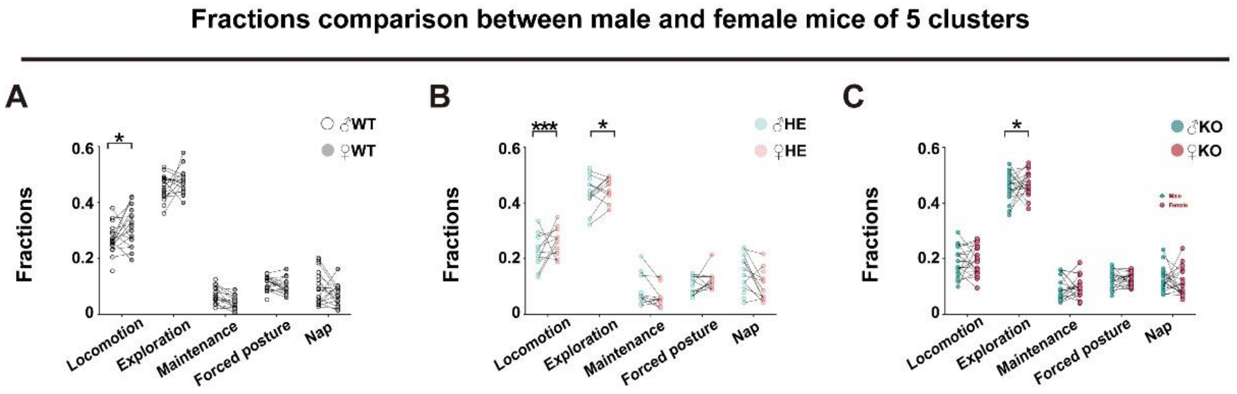

Fig. S3 Different motor characteristics between sexes of *Shank3b* WT, HE, and KO mice. **A-C** Representative Comparison of the fractions of five clusters between male and female for WT **(A)**, *Shank3b* HE **(B)**, and KO (**C**)mice. Statistics: two-way ANOVA followed by Turkey post hoc multiple comparisons test. *$P < 0.05$.

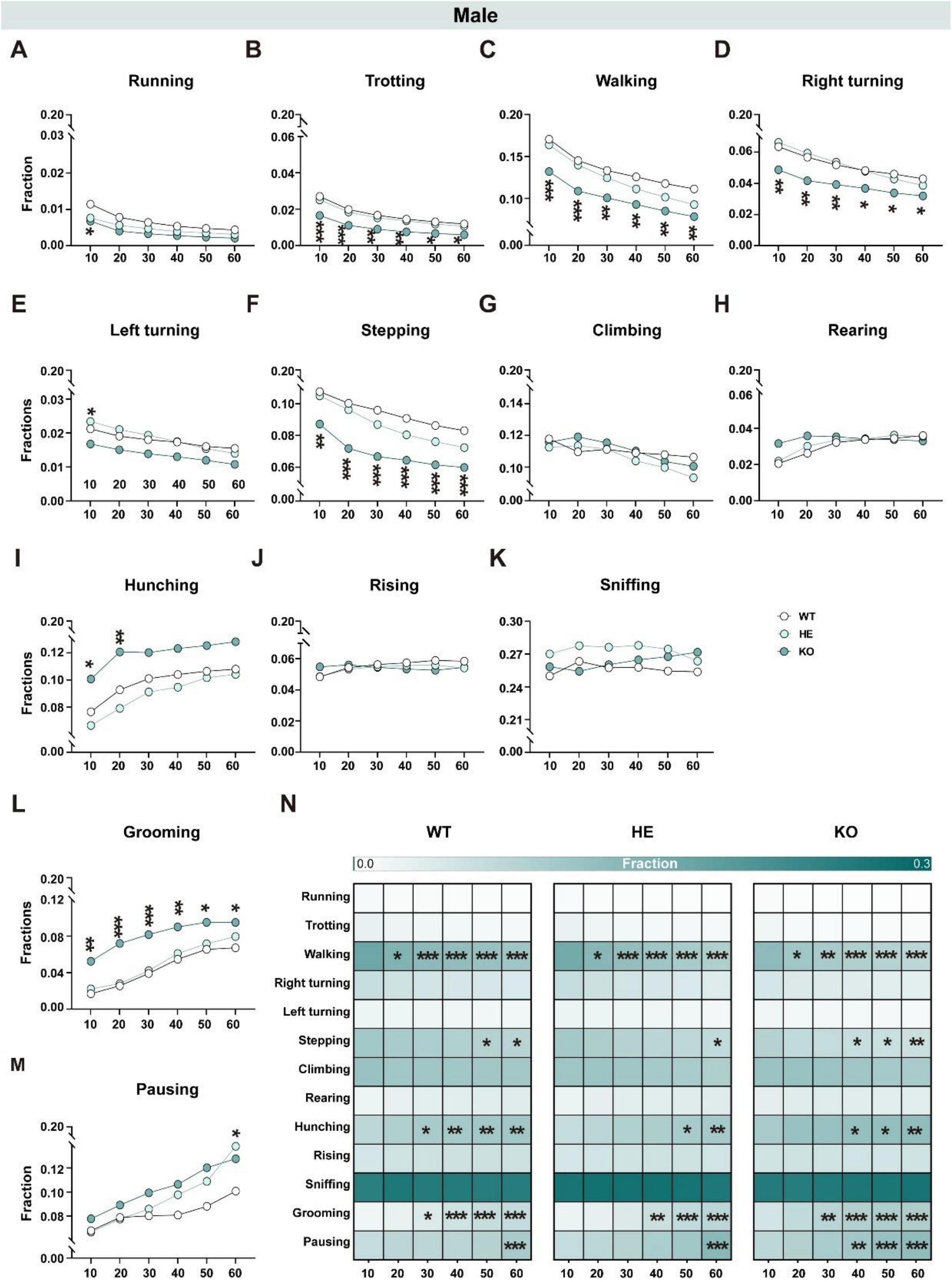

Male
A
Running
B
Trotting
C
Walking
D
Right turning
E
Left turning
F
Stepping
G
Climbing
H
Rearing
I
Hunching
J
Rising
K
Sniffing
L
Grooming
M
Pausing
N
WT
HE
KO
Fraction
Fractions
0.0
0.3
Running
Trotting
Walking
Right turning
Left turning
Stepping
Climbing
Rearing
Hunching
Rising
Sniffing
Grooming
Pausing
10 20 30 40 50 60

Fig. S4 Temporal dynamics of the thirteen movements exhibited by male *Shank3b* mutant mice. **A-M** Temporal dynamics of the thirteen movements in male *Shank3b* mutant mice (KO, deep green; HE, pale green; WT, blank) over 60 min, the sequence of the line graph representing running (**A**), trotting (**B**), walking(**C**), right turning (**D**), left turning (**E**), stepping (**F**), climbing up (**G**), rearing (**H**), hunching (**I**), rising (**J**), sniffing (**K**), grooming (**L**), pausing (**M**). Statistics: two-way ANOVA followed by Dunnett post hoc multiple comparisons test. **N** Comparison of thirteen movements in each 10-min interval for male *Shank3b* mutant mice (Left: WT; Middle: HE; Right: KO). ***$P < 0.001$, **$P < 0.01$, *$P < 0.05$.

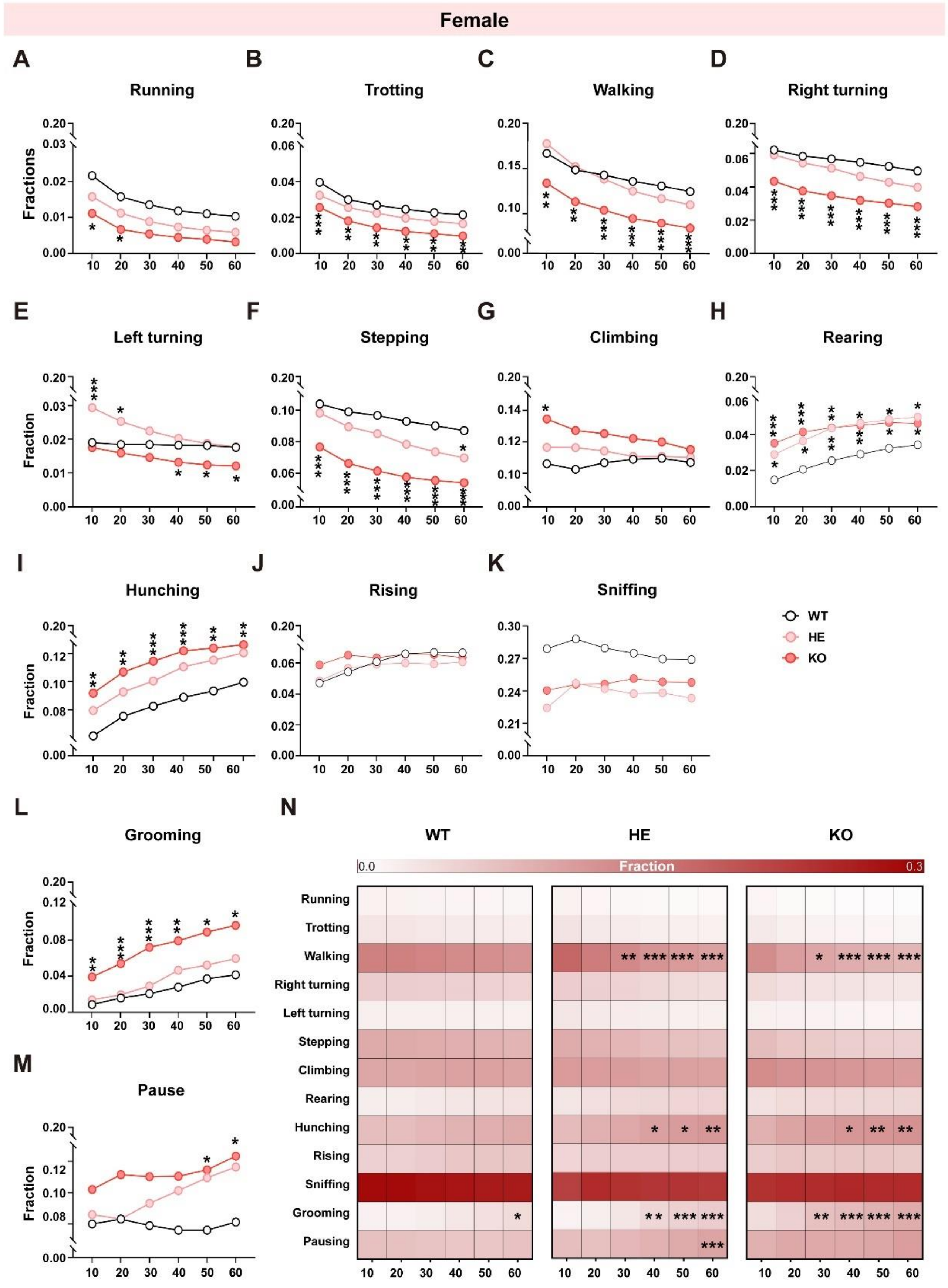

Female
A
Running
B
Trotting
C
Walking
D
Right turning
E
Left turning
F
Stepping
G
Climbing
H
Rearing
I
Hunching
J
Rising
K
Sniffing
L
Grooming
M
Pause
N
Fractions
Fraction
WT
HE
KO
Fraction
0.0
0.3
Running
Trotting
Walking
Right turning
Left turning
Stepping
Climbing
Rearing
Hunching
Rising
Sniffing
Grooming
Pausing

Fig. S5 Temporal dynamics of the thirteen movements exhibited by female *Shank3b* mutant mice. **A-M** Temporal dynamics of the thirteen movements in female *Shank3b* mutant mice (KO, deep green; HE, pale green; WT, blank) over 60 min, the sequence of the line graph representing running (**A**), trotting (**B**), walking(**C**), right turning (**D**), left turning (**E**), stepping (**F**), climbing up (**G**), rearing (**H**), hunching (**I**), rising (**J**), sniffing (**K**), grooming (**L**), pausing (**M**). Statistics: two-way ANOVA followed by Dunnett post hoc multiple comparisons test. **N** Comparison of thirteen movements in each 10-min interval for female *Shank3b* mutant mice (Left: WT; Middle: HE; Right: KO). ***$P < 0.001$, **$P < 0.01$, *$P < 0.05$.

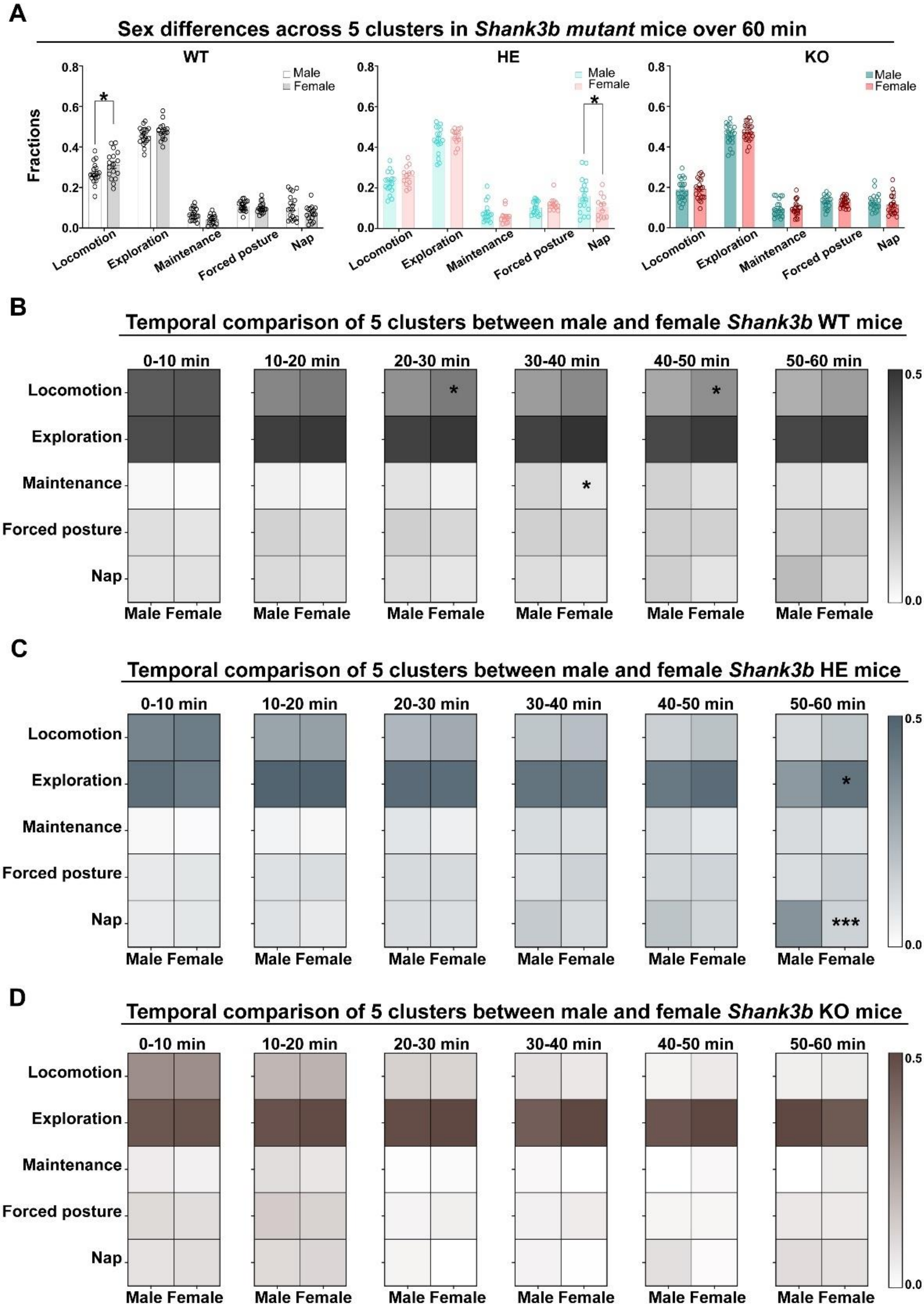
A
Sex differences across 5 clusters in Shank3b mutant mice over 60 min
WT
HE
KO
Male
Female
Fractions
Locomotion
Exploration
Maintenance
Forced posture
Nap
B
Temporal comparison of 5 clusters between male and female Shank3b WT mice
0-10 min
10-20 min
20-30 min
30-40 min
40-50 min
50-60 min
C
Temporal comparison of 5 clusters between male and female Shank3b HE mice
D
Temporal comparison of 5 clusters between male and female Shank3b KO mice

Fig. S6 Sex differences of five clusters between male and female *Shank3b* mutant mice. **A**: Comparison of the five clusters between male and female *Shank3b* WT (left), HE (middle), and KO (right) mice over a 60-minute duration, Statistics: one-way ANOVA followed by two-way ANOVA followed by Holm-Sidak post hoc multiple comparisons test. Data are expressed as mean ± SEM. **B** Temporal comparison of the five clusters in male and female *Shank3b* WT mice in every 10-min interval, with the sequence of the hot map, from left to right, respectively corresponding to 0-10 min, 10-20 min, 20-30 min, 30-40 min, 40-50 min, 50-60 min. Statistics: two-way ANOVA followed by Holm-Sidak post hoc multiple comparisons test. **C** Temporal comparison of the five clusters in male and female *Shank3b* HE mice in every 10-min interval, with the sequence of the hot map, from left to right, respectively corresponding to 0-10 min, 10-20 min, 20-30 min, 30-40 min, 40-50 min, 50-60 min. Statistics: two-way ANOVA followed by Holm-Sidak post hoc multiple comparisons test. **D** Temporal comparison of the five clusters in male and female *Shank3b* KO mice in every 10-min interval, with the sequence of the hot map, from left to right, respectively corresponding to 0-10 min, 10-20 min, 20-30 min, 30-40 min, 40-50 min, 50-60 min. Statistics: two-way ANOVA followed by Holm-Sidak post hoc multiple comparisons test. *$P < 0.05$.

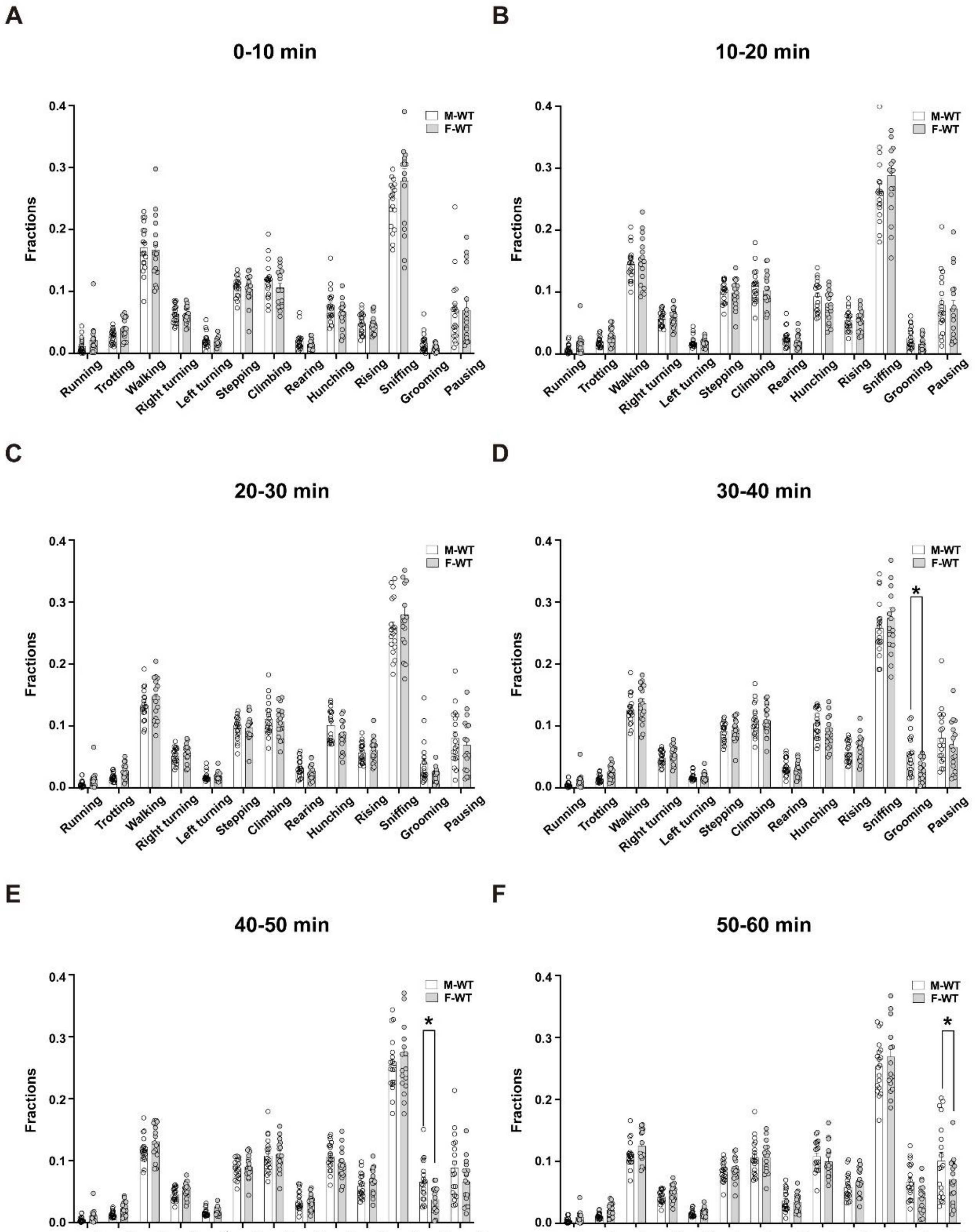
A
0-10 min
B
10-20 min
C
20-30 min
D
30-40 min
E
40-50 min
F
50-60 min
M-WT
F-WT
Fractions
0.0
0.1
0.2
0.3
0.4
Running
Trotting
Walking
Right turning
Left turning
Stepping
Climbing
Rearing
Hunching
Rising
Sniffing
Grooming
Pausing
*

Fig. S7 Temporal dynamic variations in male and female *Shank3b* WT mice. **A-F** Comparison of the thirteen movements in male and female *Shank3b* WT mice in every 10-min interval, with the sequence of the bar graph, from A to E, respectively corresponding to 0-10 min (**A**), 10-20 min (**B**), 20-30 min (**C**), 30-40 min (**D**), 40-50 min (**E**), 50-60 min (**F**). Statistics: two-way ANOVA followed by Turkey post hoc multiple comparisons test. *$P < 0.05$.

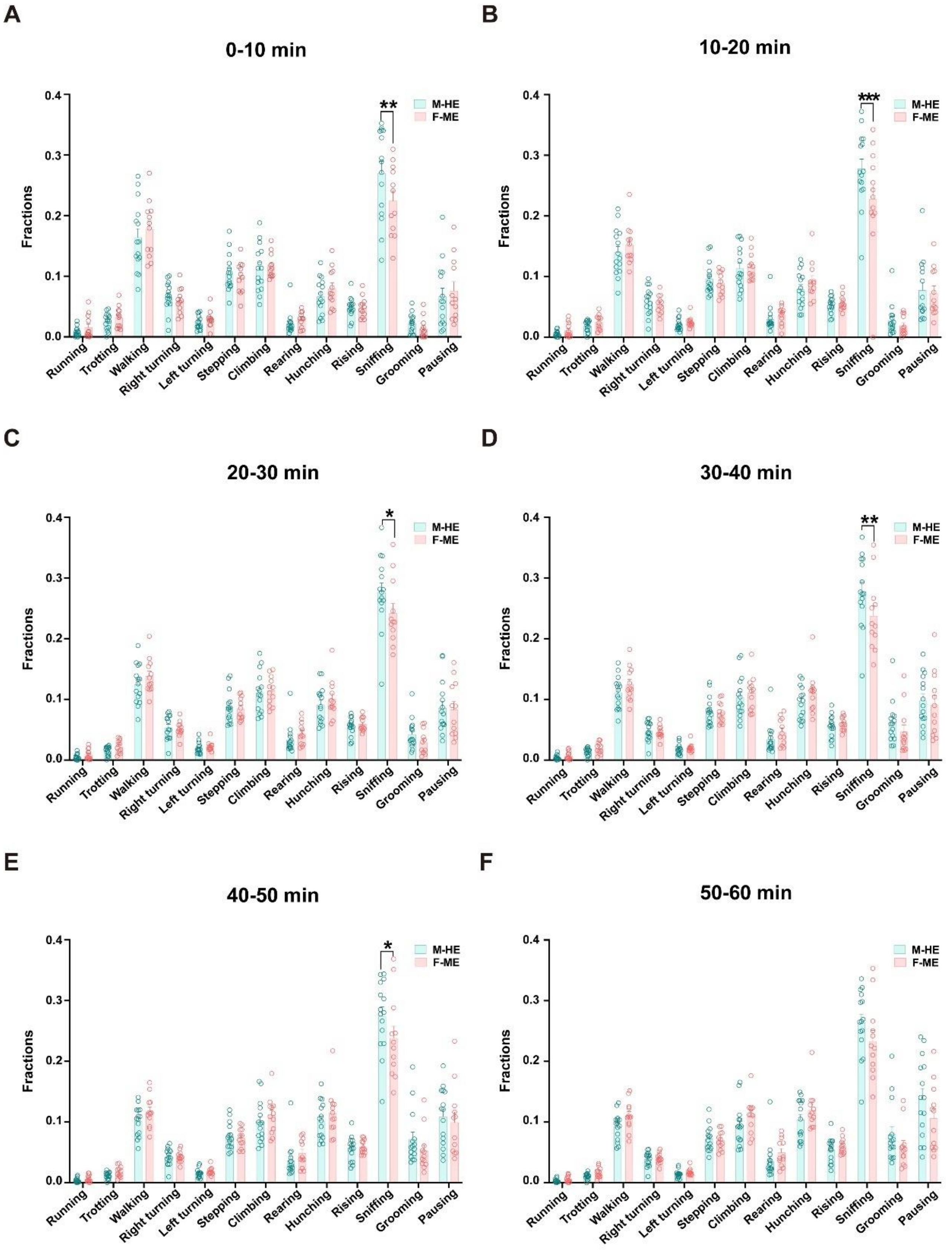

A
0-10 min
B
10-20 min
C
20-30 min
D
30-40 min
E
40-50 min
F
50-60 min
M-HE
F-ME
Fractions
0.0
0.1
0.2
0.3
0.4
Running
Trotting
Walking
Right turning
Left turning
Stepping
Climbing
Rearing
Hunching
Rising
Sniffing
Grooming
Pausing
**
***
*
**
*

Fig. S8 Temporal dynamic variations in male and female *Shank3b* HE mice. **A-F** Comparison of the thirteen movements in male and female *Shank3b* HE mice in every 10-min interval, with the sequence of the bar graph, from A to E, respectively corresponding to 0-10 min (**A**), 10-20 min (**B**), 20-30 min (**C**), 30-40 min (**D**), 40-50 min (**E**), 50-60 min (**F**). Statistics: two-way ANOVA followed by Turkey post hoc multiple comparisons test. ***$P < 0.001$, **$P < 0.01$, *$P < 0.05$.

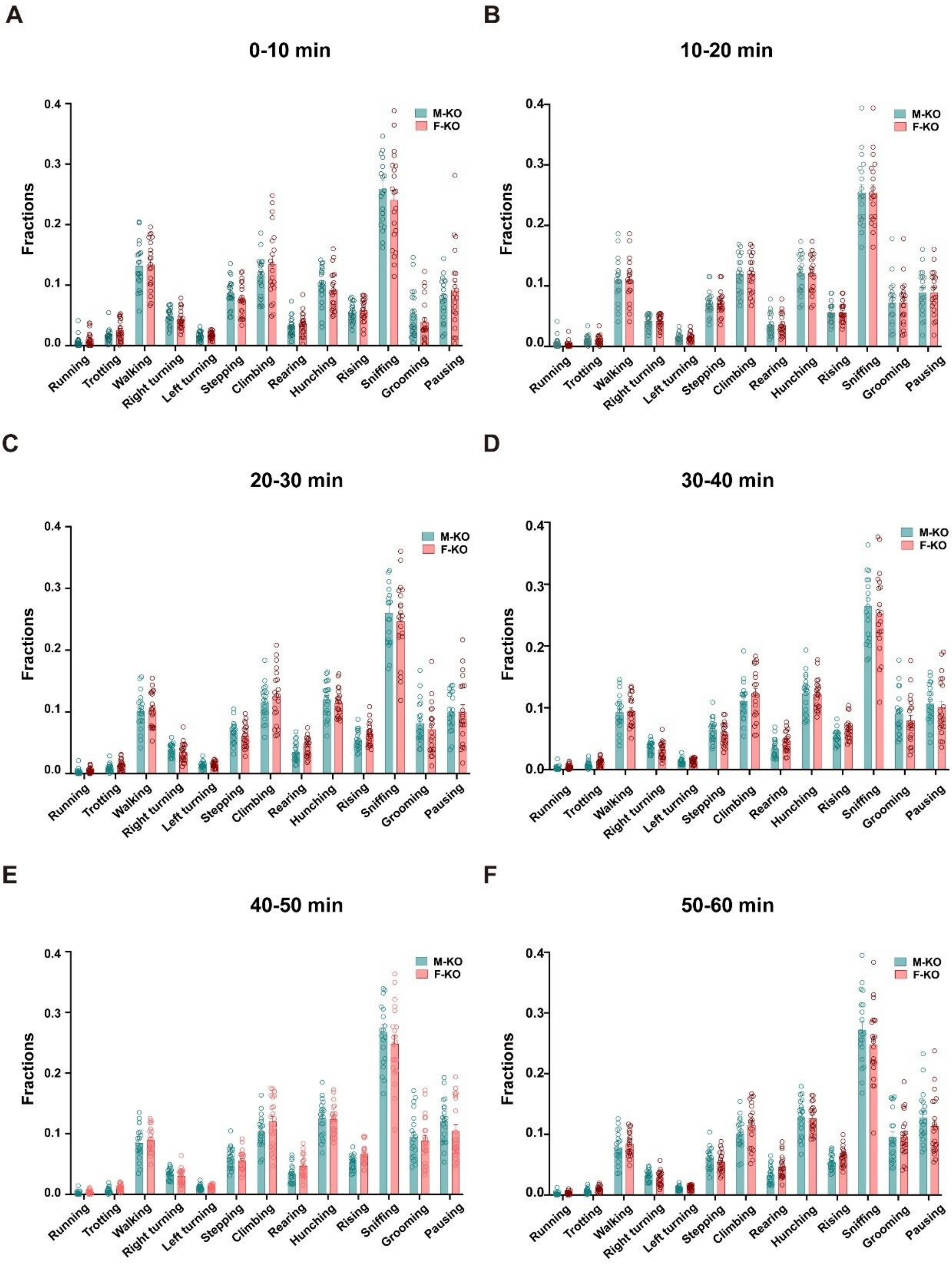
A
0-10 min
B
10-20 min
C
20-30 min
D
30-40 min
E
40-50 min
F
50-60 min
Fractions
0.0
0.1
0.2
0.3
0.4
M-KO
F-KO
Running
Trotting
Walking
Right turning
Left turning
Stepping
Climbing
Rearing
Hunching
Rising
Sniffing
Grooming
Pausing

Fig. S9 Temporal dynamic variations in male and female *Shank3b* KO mice. **A-F** Comparison of the thirteen movements in male and female *Shank3b* KO mice in every 10-min interval, with the sequence of the bar graph, from A to E, respectively corresponding to 0-10 min (**A**), 10-20 min (**B**), 20-30 min (**C**), 30-40 min (**D**), 40-50 min (**E**), 50-60 min (**F**). Statistics: two-way ANOVA followed by Turkey post hoc multiple comparisons test.

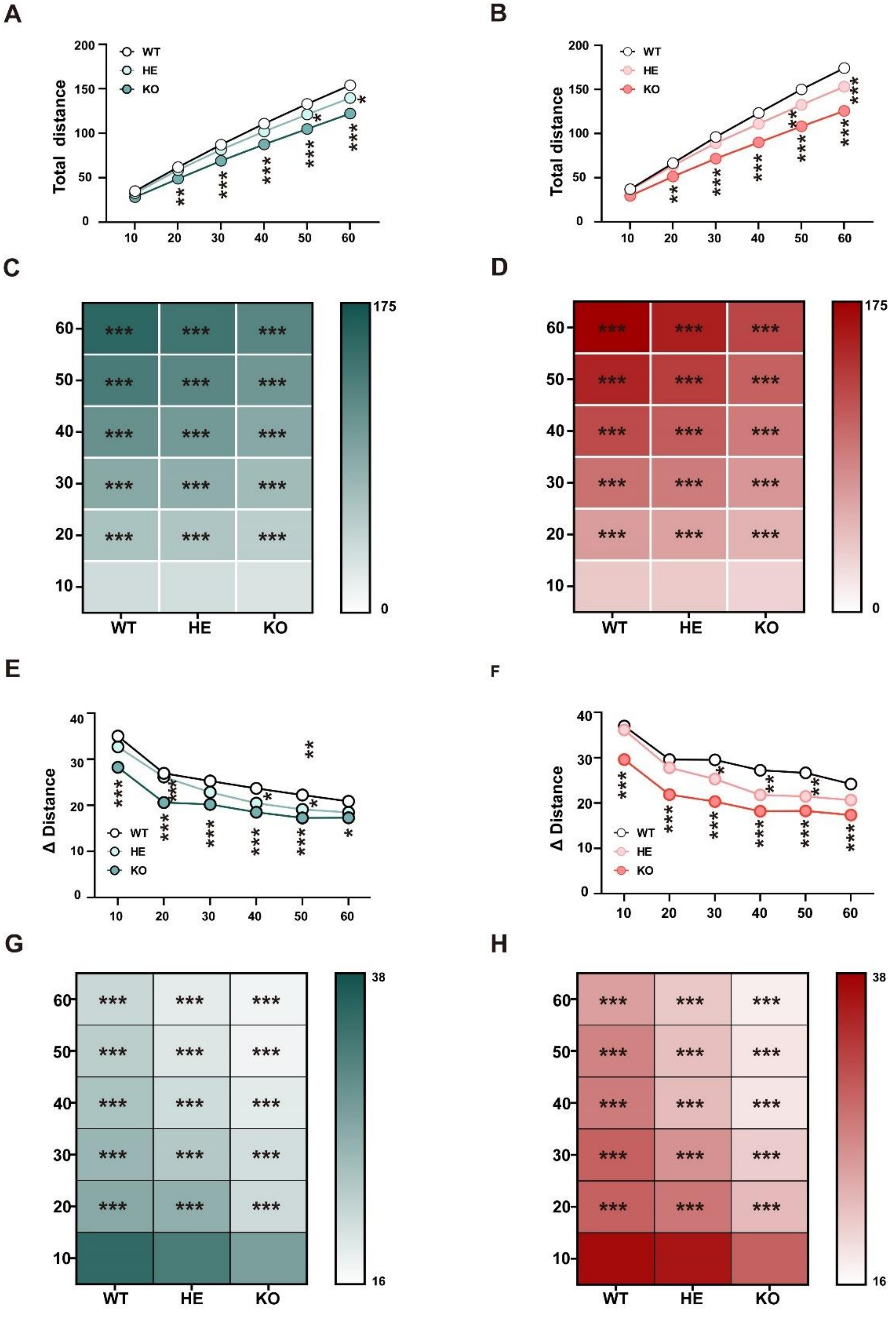

A
B
C
D
E
F
G
H
Total distance
Δ Distance
WT
HE
KO

Fig. S10 Temporal dynamics of distance traveled by male and female *Shank3b* mutant mice. **A, B, E, F** Comparative analysis of the accumulated distance (**A** for males and **B** for females) and the distance (**E** for males and **F** for females) traveled in each 10-min interval among *Shank3b* WT, HE, and KO mice. Male genotypes are depicted as follows: KO in deep green, HE in pale green, and WT in blank, while female genotypes are shown as KO in dark red, HE in pink, and WT in blank. Statistics: two-way ANOVA followed by Holm-Sidak post hoc multiple comparisons test. **C, D** Comparison of the accumulated distance traveled in each 10-min interval for male (**C**) and female (**D**) *Shank3b* WT, HE, and KO mice. Statistics: two-way ANOVA followed by Holm-Sidak post hoc multiple comparisons test. **G, H** Comparison of the distance traveled in each 10-min interval for male (**G**) and female (**H**) *Shank3b* WT, HE, and KO mice. Statistics: two-way ANOVA followed by Turkey post hoc multiple comparisons test. ***$P < 0.001$, **$P < 0.01$, *$P < 0.05$.

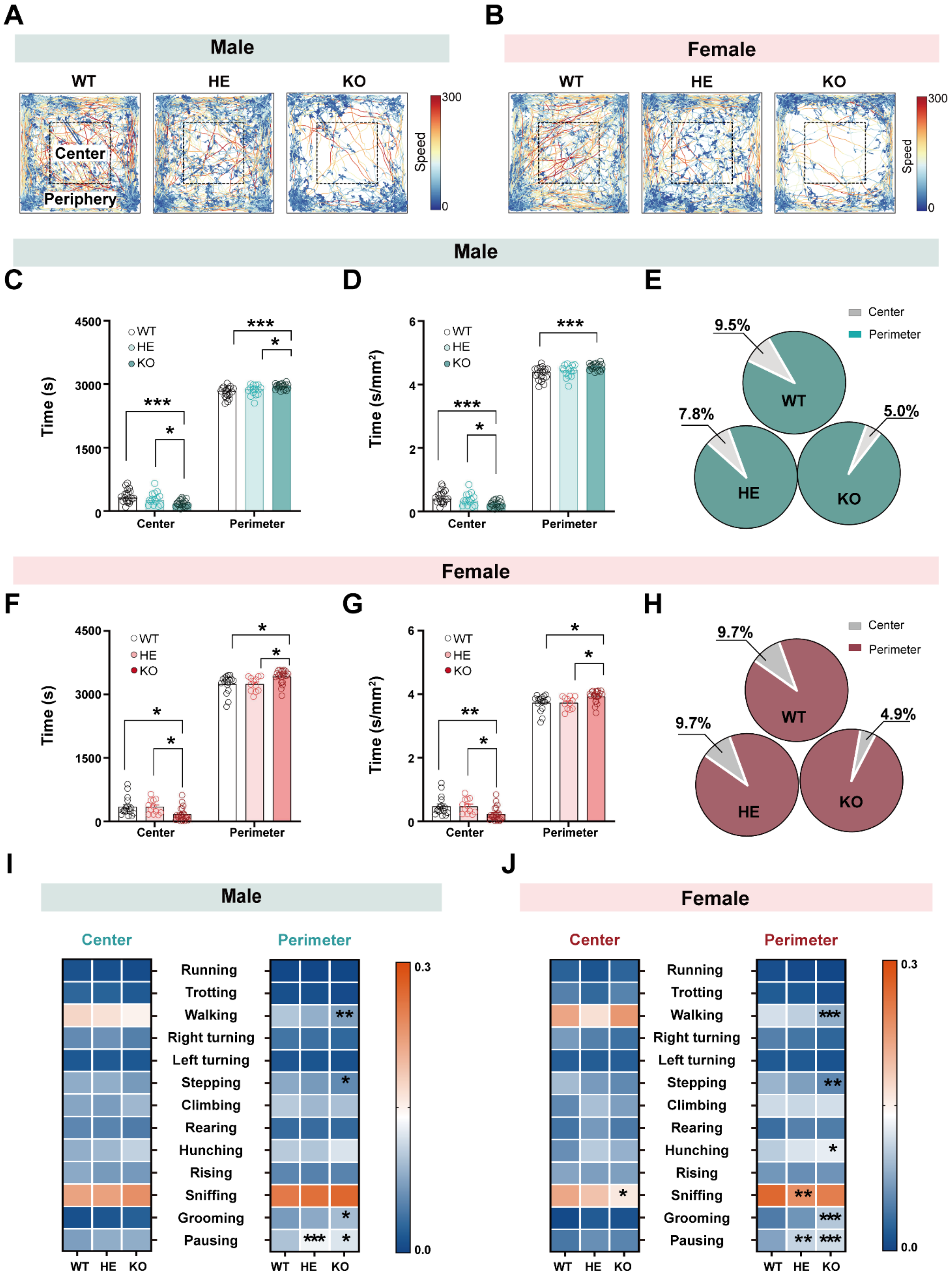
A
B
Male
Female
WT
HE
KO
Center
Periphery
Speed
300
0
C
D
E
Time (s)
Time (s/mm²)
Center
Perimeter
9.5%
7.8%
5.0%
F
G
H
9.7%
9.7%
4.9%
I
J
Running
Trotting
Walking
Right turning
Left turning
Stepping
Climbing
Rearing
Hunching
Rising
Sniffing
Grooming
Pausing
0.3
0.0

Fig. S11 Spatial distribution characteristics of movements within traditional area divisions exhibited by male and female *Shank3b* mutant mice. **A, B** Representative images of the spatial preference of male (**A**) and female (**B**) *Shank3b* mutant mice with three mice shown in each image (left: WT; Middle: HE; Right: KO). The dotted lines delineate the boundary between the center and the perimeter within traditional area divisions. **C, F** Comparison of the time fractions in the center and the perimeter among male (**C**) and female (**F**) *Shank3b* WT, HE, and KO mice in 60 min. **D, G** Comparison of the time fractions per unit area in the center and the perimeter among male (**D**) and female (**G**) *Shank3b* WT, HE, and KO mice in 60 min. **E, H** A pie diagram to compare the time percentage of the center and perimeter area for male (**E**) and female (**H**) *Shank3b* WT, HE, and KO mice. **I** Comparison of movement variation in the center (left) and perimeter (right) areas for male *Shank3b* WT, HE, and KO Mice Over a 60-min duration. **J** Comparison of movement variation in the center (left) and perimeter (right) areas for female *Shank3b* WT, HE, and KO Mice Over a 60-min duration. ***$P < 0.001$, **$P < 0.01$, *$P < 0.05$.

Table SI Statistics

Fig	Sex	Group	Time	Comparison	F value	P value
Fig 2A	Male	Walking	0-60 min	WT *vs.* KO	Interaction: F (24, 663) = 2.440, P=0.0002 Movements: F (12, 663) = 297.0, P<0.0001 Genotypes: F (2, 663) = 9.341e-006, P>0.9999	0.0012
		Stepping		WT *vs.* KO		0.0346
		Hunching		WT *vs.* KO		0.0776
		Grooming		WT *vs.* KO		0.0081
		Pausing		WT *vs.* KO		0.0110
				WT *vs.* HE		0.0006
	Female	Walking	0-60 min	WT *vs.* KO	Interaction: F (24, 611) = 5.068, P<0.0001 Movements: F (12, 611) = 236.6, P<0.0001 Genotypes: F (2, 611) = 1.820e-006, P>0.9999	<0.0001
				HE *vs.* KO		0.0344
		Stepping		WT *vs.* KO		0.0012
		Hunching		WT *vs.* KO		0.0129
		Grooming		WT *vs.* KO		<0.0001
				HE *vs.* KO		0.0012
		Pausing		WT *vs.* KO		<0.0001
				WT *vs.* HE		0.0034
Fig 3 A	Male	Locomotion	0-60 min	WT *vs.* KO	Interaction: F (8, 265) = 5.935, P<0.0001 Clusters: F (4, 265) = 514.9, P<0.0001 Genotypes: F (2, 265) = 9.969e-012, P>0.999	<0.0001
				HE *vs.* KO		0.0235
		Nap		WT vs. HE		0.0011
Fig 3 B	Female	Locomotion	0-60 min	WT *vs.* KO	Interaction: F (8, 235) = 13.43, P<0.0001 Clusters: F (4, 235) = 721.0, P<0.0001 Genotypes: F (2, 235) = 1.294e-011, P>0.9999	<0.0001
				WT *vs.* HE		0.0048
				HE *vs.* KO		<0.0001
		Maintenance		WT *vs.* KO		0.0003
				HE *vs.* KO		0.0463
		Nap		WT *vs.* KO		0.0076
Fig 3 C-G	Male	Locomotion	0-10 min	WT *vs.* KO	Interaction: F (10, 318) = 1.664, P=0.0882 Time: F (5, 318) = 72.36, P<0.0001 Genotypes: F (2, 318) = 38.43, P<0.0001	<0.0001
			10-20 min	WT *vs.* KO		<0.0001
			20-30 min	WT *vs.* KO		0.0017
			30-40 min	WT *vs.* KO		0.0035
				WT *vs.* HE		0.0179
			40-50 min	WT *vs.* KO		0.0020
				WT *vs.* HE		0.0127
			50-60 min	WT *vs.* KO		0.0051
				WT *vs.* HE		0.0024

Fig	Sex	Group	Time	Comparison	*F* value	*P* value
		Exploration	50-60 min	WT vs. HE	Interaction: *F* (10, 318) = 2.901, *P*=0.0017<br>Time: *F* (5, 318) = 3.920, *P*=0.0018<br>Genotypes: *F* (2, 318) = 2.793, *P*=0.0628	<0.0001
		Maintenance	10-20 min	WT *vs.* KO	Interaction: *F* (10, 318) = 0.7324, *P*=0.6938<br>Time: *F* (5, 318) = 11.93, *P*<0.0001<br>Genotypes: *F* (2, 318) = 4.761, *P*=0.0092	0.0199
		Nap	30-40 min	WT *vs.* HE	Interaction: *F* (10, 318) = 3.156, *P*=0.0007<br>Time: *F* (5, 318) = 17.07, *P*<0.0001<br>Genotypes: *F* (2, 318) = 9.421, *P*=0.0001	0.0223
			50-60 min	WT vs. HE		<0.0001
	Female	Locomotion	0-10 min	WT *vs.* KO	Interaction: *F* (10, 282) = 0.9411, *P*=0.4957<br>Time: *F* (5, 282) = 43.54, *P*<0.0001<br>Genotypes: *F* (2, 282) = 76.30, *P*<0.0001	<0.0001
			10-20 min	WT *vs.* KO		<0.0001
			20-30 min	WT *vs.* KO		<0.0001
			30-40 min	WT *vs.* KO		<0.0001
				WT *vs.* HE		0.0046
			40-50 min	WT *vs.* KO		<0.0001
				WT *vs.* HE		0.0083
			50-60 min	WT *vs.* KO		<0.0001
				WT *vs.* HE		0.0497
		Maintenance	10-20 min	WT *vs.* KO	Interaction: *F* (10, 282) = 0.7463, *P*=0.6804<br>Time: *F* (5, 282) = 11.80, *P*<0.0001<br>Genotypes: *F* (2, 282) = 23.04, *P*<0.0001	0.0490
			20-30 min	WT *vs.* KO		0.0003
			30-40 min	WT *vs.* KO		0.0229
				WT *vs.* HE		0.0352
			40-50 min	WT *vs.* KO		0.0180
			50-60 min	WT *vs.* KO		0.0012

Fig	Sex	Group	Time	Comparison	*F* value	*P* value
		Forced posture	10-20 min	WT *vs.* KO	Interaction: F (10, 282) = 0.4257, P=0.9336	0.0358
			20-30 min	WT *vs.* KO	Time: F (5, 282) = 12.78, P<0.0001	0.0335
			30-40 min	WT *vs.* KO	Genotypes: F (2, 282) = 11.70, P<0.0001	0.0179
		Nap	40-50 min	WT *vs.* KO	Interaction: F (10, 282) = 0.719, P=0.7057	0.0331
				WT *vs.* HE		0.0248
			50-60 min	WT *vs.* KO	Time: F (5, 282) = 3.533, P=0.0041 Genotypes: F (2, 282) = 10.87, P<0.0001	0.0168
Fig 4 C	Male	Center	0-60 min	WT vs. KO	Interaction: F (2, 102) = 15.29, P<0.0001	0.0005
		perimeter			Spatial: F (1, 102) = 3264, P<0.0001 Genotypes: F (2, 102) = 5.297e-019, P>0.9999	0.0005
Fig 4 D	Male	Center			Interaction: F (2, 102) = 15.17, P<0.0001	0.0001
		perimeter			Spatial: F (1, 102) = 2438, P<0.0001 Genotypes: F (2, 102) = 0.1195, P=0.8875	0.0017
Fig 4 *F*	Female	Center	0-60 min	WT *vs.* KO	Interaction: F (2, 94) = 10.16, P=0.0001	0.0131
				HE *vs.* KO		0.0484
		perimeter		WT *vs.* KO	Spatial: F (1, 94) = 1548, P<0.0001	0.0131
				HE *vs.* KO	Genotypes: F (2, 94) = 2.632e-019, P>0.9999	0.0484
Fig 4 G	Female	Center		WT *vs.* KO	F (2, 94) = 10.08 P=0.0001	0.0064
				HE *vs.* KO		0.0291
		perimeter		WT *vs.* KO	F (1, 94) = 1157 P<0.0001 F (2, 94) = 0.07943 P=0.9237	0.0271

Table SII Animal information

Video index	Test date	Filename	Mouse ID	Sex	Group	Test time	Recording duration	Camera type
1	20211231	rec-4-WT377MALE-20211231121633	377	Male	WT	12:16:33	60 min	RGB
2	20211231	rec-3-WT396MAEL-20211231111035	396	Male	WT	11:10:35	60 min	RGB
3	20220105	rec-1-KO376MALE-20220105095850	376	Male	KO	09:58:50	60 min	RGB
4	20220105	rec-2-WT383MALE-20220105110525	383	Male	WT	11:05:25	60 min	RGB
5	20220105	rec-4-KO384MALE-20220105131525	384	Male	KO	13:15:25	60 min	RGB
6	20220105	rec-3-KO393MALE-20220105120936	393	Male	KO	12:09:36	60 min	RGB
7	20220106	rec-1-KO385MALE-20220106094106	385	Male	KO	09:41:06	60 min	RGB
8	20220107	rec-4-WT369FEMAL-20220107104449	369	Female	WT	10:44:49	60 min	RGB
9	20220107	rec-3-WT395MALE-20220107094138	395	Male	WT	9:41:38	60 min	RGB
10	20220111	rec-4-WT389FEMAL-20220111115404	389	Female	WT	11:54:04	60 min	RGB
11	20220111	rec-3-KO406FEMAL-20220111104956	406	Female	KO	10:49:56	60 min	RGB
12	20220111	rec-1-HE413MALE-20220111083530	413	Male	HE	08:35:30	60 min	RGB

Video index	Test date	Filename	Mouse ID	Sex	Group	Test time	Recording duration	Camera type
13	20220111	rec-2-KO426MALE-20220111094447	426	Male	KO	09:44:47	60 min	RGB
14	20220112	rec-6-KO390FEMAL-20220112130742	390	Female	KO	13:07:42	60 min	RGB
15	20220112	rec-5-KO408MALE-20220112120023	408	Male	KO	12:00:23	60 min	RGB
16	20220113	rec-1-WT374FEMAL-20220113083328	374	Female	WT	8:33:28	60 min	RGB
17	20220113	rec-6-HE380FEMAL-20220113114541	380	Female	HE	11:45:41	60 min	RGB
18	20220113	rec-5-WT386FEMAL-20220113104218	386	Female	WT	10:42:18	60 min	RGB
19	20220113	rec-8-KO428FEMAL-20220113135526	428	Female	KO	13:55:26	60 min	RGB
20	20220113	rec-7-WT429FEMAL-20220113125150	429	Female	WT	12:51:50	60 min	RGB
21	20220114	rec-2-HE373FEMAL-20220114094658	373	Female	HE	09:46:58	60 min	RGB
22	20220114	rec-1-KO387FEMAL-20220114084215	387	Female	KO	08:42:15	60 min	RGB
23	20220114	rec-9-WT467MALE-20220114132802	467	Male	WT	13:28:02	60 min	RGB
24	20220114	rec-8-KO472MALE-20220114122407	472	Male	KO	12:24:07	60 min	RGB
25	20220115	rec-7-WT397MALE-20220115130415	397	Male	WT	13:04:15	60 min	RGB

Video index	Test date	Filename	Mouse ID	Sex	Group	Test time	Recording duration	Camera type
26	20220115	rec-6-WT399MALE-20220115115827	399	Male	WT	11:58:27	60 min	RGB
27	20220115	rec-4-WT427FEMAL-20220115094855	427	Female	WT	9:48:55	60 min	RGB
28	20220115	rec-5-KO471MALE-20220115105237	471	Male	KO	10:52:37	60 min	RGB
29	20220116	rec-5-KO398MALE-20220116112456	398	Male	KO	11:24:56	60 min	RGB
30	20220116	rec-7-HE410MALE-20220116133113	410	Male	HE	13:31:13	60 min	RGB
31	20220116	rec-6-WT424MALE-20220116122820	424	Male	WT	12:28:20	60 min	RGB
32	20220116	rec-2-KO430FEMAL-20220116093806	430	Female	KO	09:38:06	60 min	RGB
33	20220116	rec-1-KO431FEMAL-20220116083359	431	Female	KO	08:33:59	60 min	RGB
34	20220117	rec-7-WT332MALE-20220117124417	332	Male	WT	12:44:17	60 min	RGB
35	20220117	rec-6-WT344MALE-20220117113924	344	Male	WT	11:39:24	60 min	RGB
36	20220117	rec-1-KO351FEMAL-20220117083457	351	Female	KO	08:34:57	60 min	RGB
37	20220117	rec-2-WT388FEMAL-20220117094103	388	Female	WT	09:41:03	60 min	RGB
38	20220118	rec-1-KO335FEMAL-20220118083126	335	Female	KO	08:31:26	60 min	RGB

Video index	Test date	Filename	Mouse ID	Sex	Group	Test time	Recording duration	Camera type
39	20220118	rec-7-WT347MALE-20220118115340	347	Male	WT	11:53:40	60 min	RGB
40	20220118	rec-8-WT355MALE-20220118125642	355	Male	WT	12:56:42	60 min	RGB
41	20220118	rec-2-KO361FEMAL-20220118093550	361	Female	KO	9:35:50	60 min	RGB
42	20220119	rec-6-KO345MALE-20220119134055	345	Male	KO	13:40:55	60 min	RGB
43	20220119	rec-5-WT354MALE-20220119123722	354	Male	WT	12:37:22	60 min	RGB
44	20220121	rec-7-WT338FEMAL-20220121132310	338	Female	WT	13:23:10	60 min	RGB
45	20220121	rec-8-KO346MALE-20220121142632	346	Male	KO	14:26:32	60 min	RGB
46	20220121	rec-2-WT348FEMAL-20220121093611	348	Female	WT	09:36:11	60 min	RGB
47	20220121	rec-5-WT349FEMAL-20220121111705	349	Female	WT	11:17:05	60 min	RGB
48	20220121	rec-6-WT350FEMAL-20220121122053	350	Female	WT	12:20:53	60 min	RGB
49	20220121	rec-1-WT358FEMAL-20220121083258	358	Female	WT	08:32:58	60 min	RGB
50	20220209	rec-2-HE493FEMA-20220209110520	493	Female	HE	11:05:20	60 min	RGB
51	20220209	rec-4-HE500MALE-20220209131609	500	Male	HE	13:16:09	60 min	RGB

Video index	Test date	Filename	Mouse ID	Sex	Group	Test time	Recording duration	Camera type
52	20220209	rec-3-HE506MALE-20220209121118	506	Male	HE	12:11:18	60 min	RGB
53	20220209	rec-1-HE511FEMA-20220209094630	511	Female	HE	09:46:30	60 min	RGB
54	20220210	rec-8-HE497MALE-20220210131548	497	Male	HE	13:15:48	60 min	RGB
55	20220210	rec-7-HE504MALE-20220210115247	504	Male	HE	11:52:47	60 min	RGB
56	20220210	rec-1-HE508FEMA-20220210082733	508	Female	HE	08:27:33	60 min	RGB
57	20220210	rec-6-HE510FEMA-20220210105051	510	Female	HE	10:50:51	60 min	RGB
58	20220211	rec-1-HE490FEMA-20220211082410	490	Female	HE	08:24:10	60 min	RGB
59	20220211	rec-6-HE492FEMA-20220211104131	492	Female	HE	10:41:31	60 min	RGB
60	20220211	rec-8-HE501MALE-20220211132141	501	Male	HE	13:21:41	60 min	RGB
61	20220211	rec-7-HE507MALE-20220211114428	507	Male	HE	11:44:28	60 min	RGB
62	20220212	rec-1-HE505MALE-20220212082215	505	Male	HE	08:22:15	60 min	RGB
63	20220212	rec-6-HE512FEMA-20220212103809	512	Female	HE	10:38:09	60 min	RGB
64	20220212	rec-7-HE514FEMA-20220212114407	514	Female	HE	11:44:07	60 min	RGB

Video index	Test date	Filename	Mouse ID	Sex	Group	Test time	Recording duration	Camera type
65	20220420	rec-1-HE596MALE-20220420105258	596	Male	HE	10:52:58	60 min	RGB
66	20220420	rec-3-HE621MALE-20220420132010	621	Male	HE	13:20:10	60 min	RGB
67	20220420	rec-2-HE623MALE-20220420115514	623	Male	HE	11:55:14	60 min	RGB
68	20220421	rec-2-HE761FEMAL-20220421103537	761	Female	HE	10:35:37	60 min	RGB
69	20220421	rec-3-HE773FEMAL-20220421114703	773	Female	HE	11:47:03	60 min	RGB
70	20220421	rec-1-HE775FEMAL-20220421092747	775	Female	HE	09:27:47	60 min	RGB
71	20220422	rec-4-KO602FEMAL-20220422114544	602	Female	KO	11:45:44	60 min	RGB
72	20220422	rec-3-KO605FEMAL-20220422104153	605	Female	KO	10:41:53	60 min	RGB
73	20220422	rec-2-KO608FEMAL-20220422093436	608	Female	KO	09:34:36	60 min	RGB
74	20220422	rec-1-KO746MALE-20220422082515	746	Male	KO	08:25:15	60 min	RGB
75	20230316	rec-1-MaleKO594-20230316094745	594	Male	KO	09:47:45	60 min	RGB
76	20230316	rec-2-MaleWT595-20230316104912	595	Male	WT	10:49:12	60 min	RGB
77	20230316	rec-1-MKO759-20230316095011	759	Male	KO	09:50:11	60 min	RGB
78	20230316	rec-3-MKO767-20230316115339	767	Male	KO	11:53:39	60 min	RGB
79	20230316	rec-2-MKO768-20230316105205	768	Male	KO	10:52:05	60 min	RGB

Video index	Test date	Filename	Mouse ID	Sex	Group	Test time	Recording duration	Camera type
80	20230316	rec-3-MaleWT804-20230316115052	804	Male	WT	11:50:52	60 min	RGB
81	20230316	rec-5-MaleKO839-20230316135412	839	Male	KO	13:54:12	60 min	RGB
82	20230316	rec-4-MaleKO841-20230316125229	841	Male	KO	12:52:29	60 min	RGB
83	20230316	rec-4-MKO880-20230316125500	880	Male	KO	12:55:00	60 min	RGB
84	20230316	rec-5-MKO885-20230316135624	885	Male	KO	13:56:24	60 min	RGB
85	20230317	rec-1-MWT838-20230317101231	838	Male	WT	10:12:31	60 min	RGB
86	20230317	rec-2-MWT844-20230317111439	844	Male	WT	12:49:46	60 min	RGB
87	20230317	rec-11-FeWT883-20230317124946	883	Female	WT	12:49:46	60 min	RGB
88	20230317	rec-3-FeWT884-20230317121602	884	Female	WT	12:16:02	60 min	RGB
89	20230317	rec-12-FeKO896-20230317135120	896	Female	KO	13:51:20	60 min	RGB
90	20230317	rec-4-FeKO992-20230317131727	992	Female	KO	13:17:27	60 min	RGB
91	20230318	rec-3-FeWT716-20230318113916	716	Female	WT	11:39:16	60 min	RGB
92	20230318	rec-4-FeKO754-20230318124118	754	Female	KO	12:41:18	60 min	RGB
93	20230318	rec-5-FeKO760-20230318134233	760	Female	KO	13:42:33	60 min	RGB
94	20230318	rec-7-FeKO814-20230318111614	814	Female	KO	11:16:14	60 min	RGB
95	20230318	rec-8-FeKO846-20230318121728	846	Female	KO	12:17:28	60 min	RGB
96	20230318	rec-10-FeKO974-20230318141954	974	Female	KO	14:19:54	60 min	RGB
97	20230318	rec-9-FeKO977-20230318131844	977	Female	KO	13:18:44	60 min	RGB
98	20230318	rec-1-FeKO986-2023031809360	986	Female	KO	09:36:02	60 min	RGB
99	20230318	rec-2-FeWT987-20230318103740	987	Female	WT	10:37:40	60 min	RGB
100	20230319	rec-1-MHe979-20230319112118	979	Male	HE	11:21:18	60 min	RGB

Video index	Test date	Filename	Mouse ID	Sex	Group	Test time	Recording duration	Camera type
101	20230319	rec-10-MHe982-20230319121226	982	Male	HE	12:12:26	60 min	RGB
102	20230319	rec-2-MHe983-20230319122231	983	Male	HE	12:22:31	60 min	RGB
103	20230322	rec-5-FeWT763-20230322135711	763	Female	WT	13:57:11	60 min	RGB
104	20230322	rec-5-FeWT770-20230322135424	770	Female	WT	13:54:24	60 min	RGB
105	20230322	rec-2-MHe975-20230322103121	975	Male	HE	10:31:21	60 min	RGB
106	20230322	rec-3-MHe981-20230322113232	981	Male	WT	11:32:32	60 min	RGB
107	20230322	rec-4-MWT985-20230322125548	985	Male	WT	12:55:48	60 min	RGB
108	20230322	rec-1-MHe991-20230322092954	991	Male	HE	09:29:54	60 min	RGB

Table SIII Kinematics parameters of movements

Sex	Group	Movements	Velocity (mm)		Nose height (mm)		Back height (mm)		Horizontal angle (°)		3-dimensional angle (°)	
			Mean	Std	Mean	Std	Mean	Std	Nose-Neck-Back	Neck-Back-Tail	Nose-Neck-Back	Neck-Back-Tail
Male	WT	Running	245.74	67.93	9.06	17.74	30.13	15.19	176.99	-179.74	176.99	179.74
	HE		234.04	57.10	7.56	13.18	26.73	14.06	176.62	179.86	176.61	179.86
	KO		235.74	71.64	6.08	11.11	26.80	9.83	176.09	-179.11	176.09	179.11
Female	WT		249.26	66.91	8.52	14.17	27.93	15.92	177.31	-179.49	177.31	179.49
	HE		245.76	64.00	7.92	16.03	25.84	13.69	-177.73	-178.86	177.73	178.86
	KO		239.85	69.55	7.97	17.11	27.83	11.63	177.25	-179.59	177.25	179.59
Male	WT	Trotting	196.28	60.26	9.56	16.14	30.52	14.79	-177.83	-176.55	177.83	176.55
	HE		188.56	54.80	9.19	14.35	27.90	12.00	-177.47	-177.21	177.47	177.22
	KO		183.60	56.87	6.70	14.04	27.66	13.36	-176.28	-176.39	176.28	176.39
Female	WT		194.27	61.21	9.13	14.13	27.94	12.43	-178.16	-175.92	178.16	175.92
	HE		194.10	59.58	8.06	14.78	26.29	16.08	-177.50	-175.18	177.50	175.18
	KO		183.81	58.81	8.48	15.85	28.40	11.76	-178.24	-175.67	178.24	175.67
Male	WT	Walking	112.37	59.78	13.00	16.51	30.67	11.81	-177.90	-176.71	177.90	176.71
	HE		107.89	55.54	12.28	14.87	28.71	10.11	-177.76	-176.82	177.76	176.82
	KO		103.39	55.07	10.45	13.72	27.99	8.49	-177.76	-177.29	177.76	177.29
Female	WT		114.50	60.21	13.09	16.82	29.35	11.63	-178.36	-177.13	178.36	177.13
	HE		113.53	60.06	10.85	15.02	26.88	9.81	-178.49	-177.10	178.50	177.10
	KO		105.20	56.49	11.44	15.44	29.38	9.30	-178.51	-176.86	178.51	176.86
Male	WT	Right turning	81.08	46.97	15.01	14.77	32.27	7.61	174.13	167.17	174.13	167.17
	HE		77.23	43.95	14.22	13.77	30.70	7.34	174.43	167.07	174.43	167.07
	KO		76.05	44.65	11.65	12.58	29.17	6.81	174.07	167.81	174.07	167.81
Female	WT		81.06	47.27	15.44	15.14	31.08	8.20	174.86	167.01	174.86	167.01

Table SIII Kinematics parameters of movements

Sex	Group	Movements	Velocity (mm)		Nose height (mm)		Back height (mm)		Horizontal angle (°)		3-dimensional angle (°)	
			Mean	Std	Mean	Std	Mean	Std	Nose-Neck-Back	Neck-Back-Tail	Nose-Neck-Back	Neck-Back-Tail
	HE		79.56	47.68	13.52	14.44	29.02	8.02	175.75	166.21	175.75	166.21
	KO		76.88	46.44	13.45	13.88	30.98	8.06	175.35	167.94	175.35	167.94
Male	WT	Left turning	78.68	45.05	11.50	11.87	30.76	8.47	-178.00	-163.68	178.00	163.68
	HE		76.44	42.80	11.19	11.39	28.43	8.07	-178.52	-164.12	178.52	164.13
	KO		71.00	41.66	9.43	9.72	28.14	7.25	-178.40	-164.66	178.40	164.66
Female	WT		78.94	45.72	11.56	12.46	29.89	8.83	-177.75	-163.64	177.75	163.64
	HE		77.77	45.26	10.09	11.17	26.88	7.17	-178.01	-164.86	178.01	164.86
	KO		72.21	43.01	10.20	11.08	29.17	7.77	-178.54	-164.49	178.54	164.49
Male	WT	Stepping	50.65	28.41	18.98	16.65	33.93	9.42	-177.78	-177.56	177.78	177.56
	HE		50.25	27.07	17.97	15.30	32.63	10.02	-177.41	-177.75	177.41	177.75
	KO		50.21	27.49	16.31	15.11	31.82	9.52	-177.45	-178.04	177.45	178.04
Female	WT		50.89	29.23	19.54	17.24	33.65	11.06	-178.07	-177.44	178.07	177.44
	HE		50.59	29.01	16.97	16.06	31.04	9.61	-178.93	-176.80	178.93	176.80
	KO		50.09	28.91	17.24	15.88	33.29	10.29	-178.48	-176.26	178.48	176.26
Male	WT	Climbing	32.72	19.93	97.29	23.98	51.44	8.91	178.84	172.42	178.84	172.43
	HE		31.01	18.31	95.32	25.30	50.63	9.36	178.44	172.83	178.44	172.82
	KO		27.86	18.18	91.74	24.46	49.67	8.70	179.52	170.90	179.52	170.90
Female	WT		34.44	19.87	97.70	24.99	51.81	9.15	179.09	175.06	179.10	175.06
	HE		28.99	19.02	90.42	24.90	48.97	8.67	178.82	171.57	178.82	171.57
	KO		27.11	17.40	92.52	24.63	50.01	8.62	179.31	174.05	179.31	174.05
Male	WT	Rising	28.77	27.75	51.57	16.15	40.97	5.71	176.11	173.66	176.11	173.66
	HE		26.90	24.82	48.05	15.87	39.66	5.94	175.88	174.00	175.88	174.00

Table SIII Kinematics parameters of movements

Sex	Group	Movements	Velocity (mm)		Nose height (mm)		Back height (mm)		Horizontal angle (°)		3-dimensional angle (°)	
			Mean	Std	Mean	Std	Mean	Std	Nose-Neck-Back	Neck-Back-Tail	Nose-Neck-Back	Neck-Back-Tail
	KO		24.81	23.16	45.11	14.44	39.42	5.57	175.94	173.29	175.94	173.29
	WT		30.24	26.84	53.18	17.31	40.54	6.14	176.97	174.03	176.98	174.03
Female	HE		27.66	26.65	48.58	15.94	38.70	6.12	176.66	172.68	176.66	172.68
	KO		25.48	24.06	46.89	14.42	40.21	5.66	176.41	174.10	176.41	174.10
	WT		23.90	12.32	44.79	18.53	39.63	11.82	-174.21	-154.20	174.22	154.20
Male	HE		23.71	11.10	44.45	17.82	37.33	10.69	-173.97	-159.45	173.97	159.45
	KO		22.99	10.68	43.89	16.27	38.41	9.33	-177.39	-158.64	177.39	158.64
	WT	Grooming	24.20	10.80	47.67	16.38	40.25	14.69	-179.24	-160.55	179.24	160.55
Female	HE		22.93	10.76	50.45	17.29	38.89	10.18	-176.78	-165.30	176.78	165.30
	KO		21.44	9.67	45.34	15.45	39.73	9.56	-176.26	-153.04	176.26	153.04
	WT		23.62	17.64	48.11	18.61	39.59	7.59	178.59	179.74	178.59	179.74
Male	HE		21.95	15.36	44.42	18.59	38.74	7.99	178.25	177.75	178.25	177.75
	KO		20.20	14.28	42.38	16.35	38.76	6.89	176.23	173.59	176.23	173.59
	WT	Hunching	24.30	17.86	48.96	18.53	39.72	7.75	-178.88	-179.14	178.88	179.14
Female	HE		21.66	16.30	47.98	18.21	39.32	7.19	178.84	175.94	178.84	175.94
	KO		20.51	14.48	44.99	15.93	39.71	7.05	177.89	-179.15	177.89	179.15
	WT		22.35	13.29	80.00	16.00	45.45	6.25	-179.50	-177.68	179.50	177.68
Male	HE		22.19	12.44	78.99	17.01	44.51	6.24	-179.51	-177.72	179.51	177.72
	KO		20.56	12.69	75.03	15.72	44.66	5.98	-179.39	-178.22	179.39	178.22
	WT	Rearing	23.40	13.28	82.72	15.85	46.04	6.13	-179.48	-179.31	179.48	179.32
Female	HE		20.88	12.11	78.05	16.77	44.69	5.95	-179.57	-179.32	179.57	179.32
	KO		19.82	11.63	74.23	15.34	44.31	6.22	-179.25	-175.36	179.25	175.36

Table SIII Kinematics parameters of movements

Sex	Group	Movements	Velocity (mm)		Nose height (mm)		Back height (mm)		Horizontal angle (°)		3-dimensional angle (°)	
			Mean	Std	Mean	Std	Mean	Std	Nose-Neck-Back	Neck-Back-Tail	Nose-Neck-Back	Neck-Back-Tail
Male	WT	Sniffing	22.81	14.27	21.69	15.32	34.15	7.43	177.32	179.43	177.33	179.43
	HE		22.64	13.99	18.97	14.27	32.49	8.07	177.21	178.79	177.21	178.79
	KO		21.59	13.83	18.43	13.59	32.57	7.69	176.60	178.01	176.60	178.01
Female	WT		22.66	14.52	21.98	15.79	33.20	7.90	177.18	178.48	177.18	178.48
	HE		22.44	14.11	19.46	15.26	31.37	8.00	178.22	178.69	178.22	178.69
	KO		21.22	13.97	19.36	14.31	33.56	8.00	177.79	179.49	177.79	179.49
Male	WT	Pausing	20.34	14.44	24.54	17.37	41.28	17.76	-175.59	-160.06	175.59	160.06
	HE		21.54	13.45	18.86	19.45	39.32	20.17	-179.03	-162.73	179.03	162.73
	KO		20.24	13.68	21.92	17.83	39.46	16.75	-178.72	-165.36	178.72	165.36
Female	WT		21.45	15.82	25.65	20.05	41.76	24.36	-177.26	-161.06	177.26	161.06
	HE		20.82	14.50	22.79	22.26	40.20	20.68	-178.54	-159.07	178.54	159.07
	KO		19.33	12.88	23.05	18.20	40.39	18.17	-177.21	-159.51	177.21	159.51

Note: The horizontal angle below 0 means toward the left.
